# Recent Successes and Future Directions in Immunotherapy of Cutaneous Melanoma

**DOI:** 10.3389/fimmu.2017.01617

**Published:** 2017-12-08

**Authors:** Hassan Sadozai, Thomas Gruber, Robert Emil Hunger, Mirjam Schenk

**Affiliations:** ^1^Institute of Pathology, Experimental Pathology, University of Bern, Bern, Switzerland; ^2^Department of Dermatology, University Hospital Bern, Bern, Switzerland

**Keywords:** melanoma, immunotherapy, immune checkpoint blockade, tumor microenvironment, adoptive T cell transfer, programmed cell death protein 1, tumor-infiltrating lymphocyte, tumor-infiltrating dendritic cell

## Abstract

The global health burden associated with melanoma continues to increase while treatment options for metastatic melanoma are limited. Nevertheless, in the past decade, the field of cancer immunotherapy has witnessed remarkable advances for the treatment of a number of malignancies including metastatic melanoma. Although the earliest observations of an immunological antitumor response were made nearly a century ago, it was only in the past 30 years, that immunotherapy emerged as a viable therapeutic option, in particular for cutaneous melanoma. As such, melanoma remains the focus of various preclinical and clinical studies to understand the immunobiology of cancer and to test various tumor immunotherapies. Here, we review key recent developments in the field of immune-mediated therapy of melanoma. Our primary focus is on therapies that have received regulatory approval. Thus, a brief overview of the pathophysiology of melanoma is provided. The purported functions of various tumor-infiltrating immune cell subsets are described, in particular the recently described roles of intratumoral dendritic cells. The section on immunotherapies focuses on strategies that have proved to be the most clinically successful such as immune checkpoint blockade. Prospects for novel therapeutics and the potential for combinatorial approaches are delineated. Finally, we briefly discuss nanotechnology-based platforms which can in theory, activate multiple arms of immune system to fight cancer. The promising advances in the field of immunotherapy signal the dawn of a new era in cancer treatment and warrant further investigation to understand the opportunities and barriers for future progress.

## Metastatic Melanoma

Malignant melanoma is a highly aggressive cancer and accounts for the majority (60–80%) of deaths from skin cancer (1, 2). Non-melanoma skin cancers, including basal cell carcinomas and squamous cell carcinomas, have much lower metastatic potential and associated mortality than melanoma (3). Melanoma arises from pigment-producing cells called melanocytes that are found primarily in the skin and the eyes and to a lesser extent, in a wide range of body tissues (2, 4, 5). Melanocytes originate from the embryonic neural crest and migrate to the epidermis where they mature and produce melanin that is subsequently transferred to neighboring keratinocytes (6, 7). Melanin plays a crucial role in protecting the skin from ultraviolet (UV) solar radiation (6, 8). Neoplasia of melanocytes varies from benign melanocytic naevi to malignant melanomas (4, 5). Malignancies can arise from any of the tissues where melanocytes are present but by far the most common type is cutaneous melanoma, comprising over 90% of all melanoma cases (5, 9). Hence, the central focus of this review will be on cutaneous melanoma. Due to the recent advances in tumor immunotherapy, a number of novel cancer treatment strategies have emerged. As such, this review will discuss the development of cancer immunotherapy in the context of melanoma and highlight potential avenues for further research.

### Epidemiology

Melanoma is a fairly common cancer with an estimated global incidence rate of 3 per 100,000 (9–11). In 2015, it was reported that there were approximately 352,000 new cases of melanoma worldwide with an age-standardized incidence rate of 5 cases per 100,000 persons (12). There were nearly 60,000 deaths worldwide due to melanoma (12). The incidence rate is observed to be higher in males than in females and is associated with a younger median age (~57 years) at diagnosis than other solid tumors (~65 years) (9, 10, 12). The three regions with the highest incidence of melanoma were found to be Australasia (54%), North America (21%), and Western Europe (16%) (12). Furthermore, it is particularly concerning that the global incidence rates of melanoma continue to rise. In 2005, there were roughly 225,000 new cases of melanoma but in 2015, that number climbed to roughly 352,000 cases, representing a 56% increase (13). A large-scale cohort study from 39 countries showed that while incidence rates for melanoma are beginning to stabilize in North America and Australia, they are continuing to rise in Southern and Eastern Europe (11). Therefore, melanoma constitutes a significant burden of disease worldwide and warrants both novel treatments and prevention strategies.

### Pathophysiology and Clinical Subtypes

The exact etiology of melanoma development is not well understood (4). However, there has been tremendous study on the histological and molecular profiles of the various subtypes of melanoma (14–16). Overall, it has been observed that melanomas which arise from skin that is chronically sun-damaged (CSD) occur in anatomical locations such as the head and neck. By contrast, non-CSD melanomas are found in anatomical regions that suffer only limited sun exposure such as the trunk and extremities (4). Overall, non-CSD melanomas also have lower mutational loads than CSD melanomas (4, 16). A significant number of melanomas are usually associated with benign neoplasms of melanocytes. These lesions are termed naevi (commonly called moles), and an increased presence of naevi is deemed a risk factor for melanoma (2, 4). These lesions include benign naevi, dysplastic naevi, which display atypical cellular characteristics, and non-invasive melanoma *in situ* (4, 17). Melanoma *in situ* is by definition confined to the epidermis and if resected entirely, has a 100% survival rate (17). The current staging system for melanoma is the one used by the American Joint Committee on Cancer (AJCC) and relies upon analysis of the tumor (T), the number of metastatic nodes (N), and the presence of distant metastases (M) (18, 19). These are then grouped to provide clinical stages of the cancer, ranging from 0 to stage IV (19). Stage IV melanoma is classified as metastatic melanoma due to the presence of distant metastases, while stage III is only marked by metastases in regional lymph nodes (LN) (20).

**Figure 1:**
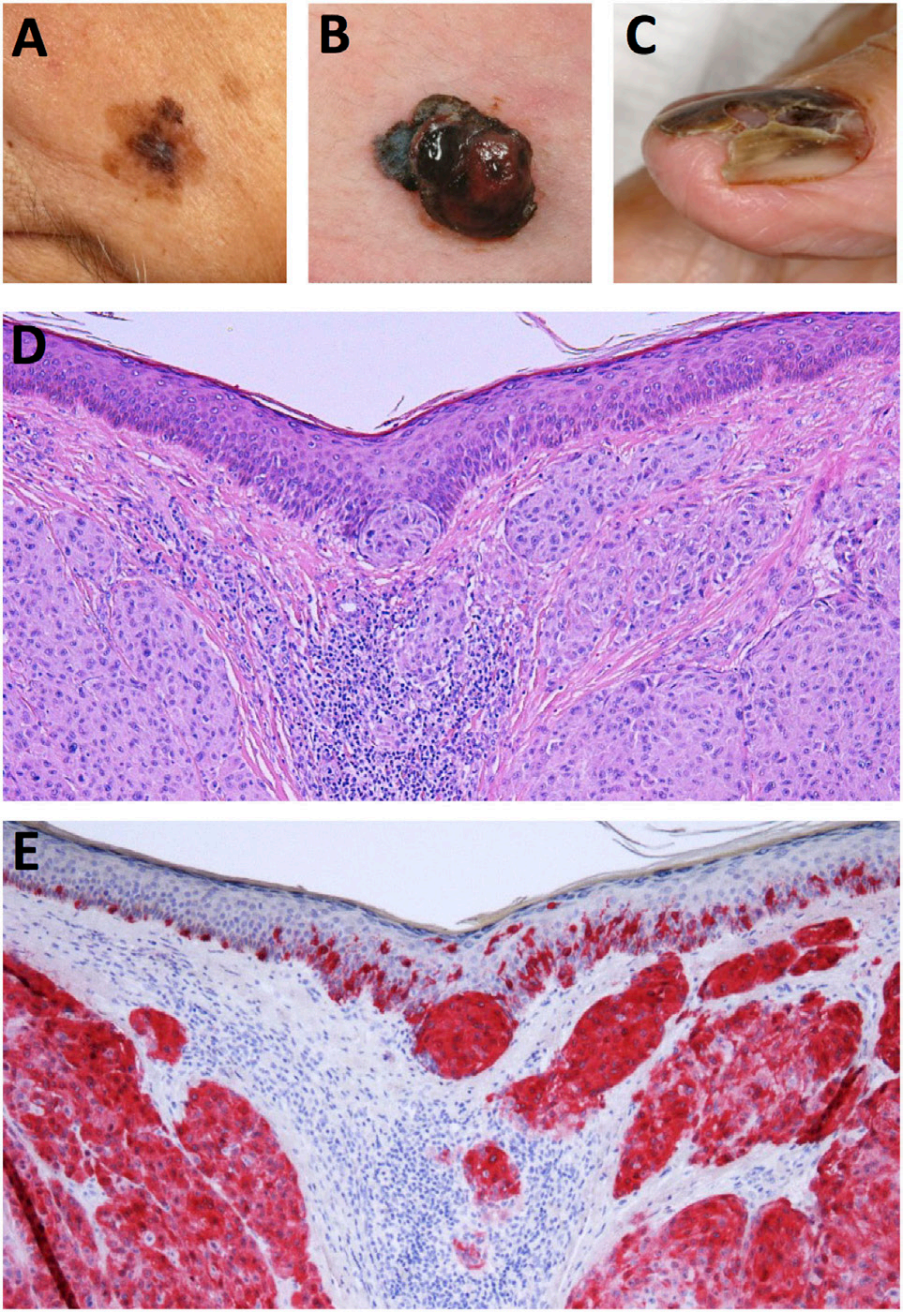
Clinical and histological presentation of melanoma. **(A)** Superficial spreading melanoma (SSM), **(B)** nodular melanoma (NM), **(C)** acrolentiginous melanoma (ALM), **(D)** H&E stain of NM depicting asymmetrical nodular tumor infiltrates in the upper dermis. Nests of atypical cells are visible in the dermis and at the dermoepidermal junction. **(E)** Immunohistochemical staining for Melan-A reveals red stained atypical tumor cells in the dermis and epidermis (Images courtesy of RH).

Historically, malignant melanoma was divided into four major histological subtypes but due to the complexity of the disease, a fraction of melanomas cannot be completely classified into either subtype (15, 21, 22). Moreover, as this classification system is reliant on clinical and morphological features, it yields little prognostic value but serves as a useful strategy in identifying the various histological forms of the disease (22). The four primary subtypes of melanoma are as follows: (i) superficial spreading melanoma (SSM), (ii) nodular melanoma (NM), (iii) lentigo maligna melanoma (LMM), and (iv) acral lentiginous melanoma (ALM) (14, 22). However, in recent years, a number of novel clinical subtypes have also been defined. These include desmoplastic melanoma (DM), melanoma arising from a blue naevus and persistent melanoma (22). The five common histogenic subtypes of melanoma warrant further description here. A pictorial overview of the clinical manifestation and histopathology of melanoma is presented in Figure 1.

#### Superficial Spreading Melanoma

Superficial spreading melanomas are the most common subtype representing between 50 and 70% of all cases (14, 23). They occur in relatively younger patients (~50 s) and present on anatomical regions such as the trunk, back, and extremities (22). SSM presents as a flat or a slightly elevated lesion with varying pigmentation (24). Histologically, SSM is marked by atypical melanocytes with nested or single cell upward migration (22). Malignant melanocytes display lateral spreading throughout the epidermis, poor circumscription, and increased melanization in the cytoplasm (14, 22).

#### Nodular Melanoma

Nodular melanomas are a fairly common subtype of melanoma (15–35%) that can present most commonly on the head and neck as a growing nodule that shows ulceration (22–24). Histologically, NMs show similarities to SSMs but differ in that they show distinct circumscription. They do not display radial growth but aggressive vertical growth evidenced by large dermal nests and sheets of atypical melanocytes (14, 22).

#### Lentigo Maligna Melanoma

Lentigo maligna melanomas present almost exclusively on the sun-exposed upper extremities or head and neck of elderly people (mostly octogenarians) (22). It is relatively uncommon (5–15%), and topically can be seen as patch of discolored skin showing variegated coloring (23, 24). Lentigo maligna (Hutchinson’s freckle) is the term for the *in situ* melanoma phase, and a small percentage of these patients progress to invasive LMM (23). Histologically, the skin exhibits extensive solar damage resulting in an atrophic epidermis and lentiginous (back-to-back) proliferation of melanocytes, which are hyperchromatic (22). Multinucleated (starburst form) melanocyte cells and solar elastosis are also hallmarks of this type of melanoma (14).

#### Acral Lentiginous Melanoma

Acral lentiginous melanomas are a fairly uncommon subtype (5–10%) and occur primarily in non-Caucasian populations such as people of African or Japanese descent (23). They present on acral sites such as palms, soles of the feet, or under the nails. On the skin they present as slow growing patches with variegated pigmentation (22). Histologically, this subtype displays single cells or nests of melanocytes along the dermal–epidermal junction, and the association of lymphocyte infiltrates can be used as a diagnostic marker for this subtype of melanomas (14, 22).

#### Desmoplastic Melanoma

Desmoplastic melanoma is a rare form of melanoma comprising 4% of primary melanomas and defined by the histological features observed in its dermal component (22, 25). It occurs primarily on the head and neck region in elderly individuals and is associated with higher probability of recurrence but a lower incidence of metastasis (25). Histologically, it is characterized by spindle-shaped melanocytes and a desmoplastic stroma, i.e., new collagen formation, and usually appears to be amelanotic (22, 25).

### Risk Factors and Driver Mutations

Melanoma occurs via a complex interplay of genetic and environmental risk factors. The primary environmental risk factor of concern is UV solar radiation as well as, UV rays from tanning beds (26, 27). Individual risk factors include the increased presence of melanocytic naevi, skin complexion, and in certain cases, family history of melanoma (26, 28). Melanomas display one of the highest mutational burdens among solid tumors (25). Thus, the molecular profiles that are associated with various subtypes of melanoma are the subject of current studies. In particular, it is crucial to distinguish “driver” mutations, or mutations that confer a survival advantage, from “passenger” mutations, which have negligible or no contribution to tumor growth (29). Understanding the mutational landscapes of a cancer allows for the development of targeted therapies that can significantly improve clinical outcomes. A massive study conducted by researchers of The Cancer Genome Atlas Network, was reported in 2015, and determined the first-ever comprehensive genomic classification system for cutaneous melanomas (30). These four distinct subtypes were based on the pattern of the major significantly mutated genes, i.e., BRAF, RAS, neurofibromin 1 (NF1), and triple wild type (WT), which denotes a lack of mutations in the three aforementioned genes but is associated with higher copy number and structural rearrangement abnormalities. These subtypes do not correlate with outcome but may help delineate the genomic changes associated with melanoma thereby providing potential molecular targets (30). Of further interest was the observation that immune gene expression, and immune cellular infiltrates did correlate with patient survival (30). As the studies of the major genomic aberrations in melanoma have been extensively reviewed elsewhere, this section will describe a number of the most common driver mutations seen in cutaneous melanoma [BRAF, NRAS, NF1, microphthalmia-associated transcription factor (MITF), and PTEN] (4, 15, 25, 28, 31).

#### BRAF

Nearly 60% of melanoma cases have mutations in BRAF (v-raf murine sarcoma viral oncogene homolog B) (25, 32). Thus, a brief overview of BRAF signaling is warranted. *BRAF* codes for a serine/threonine protein kinase constituting part of the RAS–rapidly accelerated fibrosarcoma (RAF)–mitogen-activated protein kinase kinase (MEK)–extracellular signal-regulated kinase (ERK) [mitogen-activated protein kinase (MAPK)] pathway, which is activated by the binding of extracellular growth factors to receptor tyrosine kinases (32). This binding leads to the activation of RAS (named for *Rat sarcoma*) family of GTPases (proteins that bind and hydrolyze guanosine triphosphate to guanosine diphosphate, i.e., GTP to GDP), which recruit and activate RAF serine/threonine protein kinases, which in turn activate MEK resulting finally in the phosphorylation of ERK (32–35). The activation of ERK leads to downstream signaling and activation of transcription factors that mediate cell differentiation, growth, and inhibit cell death (33, 36).

BRAF is one of three mammalian RAF isoforms, and one that has the highest basal kinase activity and thus is the most common isoform mutated in human cancers that include melanoma but also hairy cell leukemia, papillary thyroid cancer and colorectal cancer (CRC) (33, 36). The missense mutation, V600E, results in a substitution from valine to glutamic acid at the 600th amino acid position and represents the majority (80%) of all BRAF activating mutations in melanoma (25, 28). Other BRAF mutations include V600K (valine–lysine) and V600R (valine–arginine). BRAF-activating mutations result in constitutively active MEK signaling leading to tumor progression. *In vitro*, the V600E mutation confers 500-fold higher activity in BRAF than normal and promotes the transformation of melanocytes to melanoma (37). BRAF^V600E^ mutations are also found in benign naevi indicating that alone, these mutations may not be sufficient for tumor progression (38). The presence of these mutations has led to the development and approval of two BRAF inhibitors (BRAFi) for melanoma treatment, namely, vemurafenib (Genentech/Plexxikon) and dabrafenib (GlaxoSmithKline) as well as, a MEK inhibitor trametinib (GSK) (33, 39).

#### NRAS

The second most common type of driver mutations in melanomas occur in NRAS (neuroblastoma RAS viral v-ras oncogene) and are found in 15–20% of melanoma patients (28). The most common mutation in NRAS occurs at codon 61 resulting in the replacement of glutamine by lysine or arginine, thereby resulting in a constitutively active RAS (38). This leads to upregulation of both the MAPK and phosphatidylinositol 3′ kinase (PI3K) pathways and results in increased cell proliferation and invasiveness (25). NRAS mutant melanomas have increased thickness and display high rates of mitosis (25). NRAS mutations are also found in benign congenital nevi (28). NRAS and BRAF activations rarely occur in the same melanoma, albeit NRAS mutations being observed in patients with advanced BRAF tumors who had failed BRAFi therapy and which therefore may mechanistically contribute to resistance to BRAFi treatment (28). Efforts to target NRAS have focused on downstream inhibitors for the MAPK pathway and include the MEK inhibitor binimetinib, which is undergoing clinical trials (25).

#### Neurofibromin 1

Neurofibromin 1 encodes a large protein of more than 2,800 amino acids with multiple functional domains (40). It contains several functional domains with one domain bearing resemblance to the catalytic region of GTPase-activating protein. This is the most well-characterized domain of NF1 and acts as a negative regulator for RAS by converting the active RAS-GTP to the inactive RAS-GDP, thus playing the role of a tumor suppressor gene (40, 41). Germline mutations in NF1 lead to a genetic syndrome called neurofibromatosis type 1 (NF1), a relatively frequent genetic condition with an incidence of 1 in 3,000, resulting in a higher predisposition to multiple tumors arising from various cell types (40). The incidence of melanoma in patients with neurofibromatosis type 1 is very low. However, NF1 somatic mutations are found in a range of cancers, and it is the third common driver mutation in melanoma found in nearly 14% of tumors (25, 41). Mutations in NF1 are more commonly observed on skin with chronic UV exposure and in elderly patients (40). NF1 inactivating mutations were found in 48% of a cohort of wild-type BRAF and NRAS melanomas and are often associated with mutations in other RAS-related genes such as RAS p21 protein activator 2 (RASA2), PTPN11, and SPRED1 (25, 40). Recent studies have also shown that NF1 may be a unique driver mutation in DMs as NF1 loss-of-function in DM is more common than for other histogenic subtypes (25). Due to the crucial role of NF1 upstream of RAS/MAPK and PI3K/mTOR pathways, NF1 mutant tumors have been targeted with tyrosine kinase inhibitors (e.g., imatinib), MEK inhibitors (trametinib), and mTOR inhibitors (sirolimus), but to date, none of these agents have been reported in treatment of NF1 mutant melanomas (40).

#### Microphthalmia-Associated Transcription Factor

Microphthalmia-associated transcription factor is a helix-loop-helix leucine zipper transcription factor required for differentiation, proliferation, and survival of melanocytes and thus, its expression is also necessary for melanoma survival (42, 43). MITF also plays an important antiapoptotic function in melanoma cells by activating the expression of genes such as *BLC2A1, BCL2*, and *BIRC7* (43). MITF is observed to be amplified in 20% of metastatic melanomas and is associated with poor survival (25). MITF is regulated by the MAPK pathway and in particular, BRAF^V600E^ causes induction of MITF through the transcription factor BRN2 (N-Oct-3) (25). Alternately, increased ERK signaling can also target MITF for degradation (44). Finally, MITF is also purported to contribute to BRAFi resistance through the regulation of the *BCL2A1* antiapoptotic gene (44). Although targeting of MITF directly may not be viable, the use of histone deacetylase (HDAC) inhibitors can reduce MITF expression. Hence, the HDAC inhibitor panobinostat in combination with decitabine and chemotherapy is being studied in clinical trials for metastatic melanoma treatment (25).

#### PTEN

Phosphatase and tensin homolog (*PTEN*) is a commonly mutated gene in melanoma and PTEN mutations were found in 14% of all melanoma samples from the TCGA genome classification study mentioned above (25, 30). *PTEN* codes for a phosphatase which targets phosphatidylinositol (3,4,5)-triphosphate and thus plays a crucial role in the aforementioned PI3K–Akt pathway (45). PTEN silencing therefore results in dysregulated apoptosis, cell cycle progression and migration, contributing to tumorigenesis (25, 45). It has been observed that *PTEN* mutations are more frequent in metastatic melanomas as opposed to early stage primary tumors (25). The loss of PTEN also interferes with genetic stability, thus sensitizing PTEN-deficient cells to polyadenosine diphosphate ribose polymerase (PARP) inhibitors (46). Currently, there are no PARP inhibitor trials underway for the treatment of metastatic melanoma (46).

### Current Treatments for Malignant Melanoma

The multiple clinical approaches to the treatment of early and advanced melanoma are reviewed elsewhere (18, 20, 47). As previously mentioned, the median survival associated with metastatic melanoma (stage IV) remains very poor, and the 10-year survival for all patients is under 10% (47). Melanoma treatments involve the use of surgery, radiation or systemic therapy (which includes immunotherapy) (18, 20). For most primary melanomas, surgical excision of the tumor remains the standard-of-care therapy. Biopsy and histological examination of the sentinel LN is an important component of melanoma staging and has been found to be a strong prognostic measure (18, 20). When surgical excision is not an option, primary lentigo maligna may also be treated with radiation or cryotherapy (20). The treatment modalities for metastatic melanoma are more complex as most single or even combination therapies are only successful in a subset of patients (18, 48). For patients with oligometastatic disease, surgery remains a primary treatment (18, 48). Melanoma is considered a relatively radiation-resistant cancer type, but radiation therapy continues to be utilized for patients with brain metastases (47, 48). Systemic therapy includes chemotherapy, targeted therapy, and immunotherapy (18, 47). Studies with various agents, including combination chemotherapy approaches, have shown that it has limited efficacy in melanoma (18, 47). The major chemotherapy drugs that have been used to treat melanoma including the alkylating agents dacarbazine, temozolomide, and nitrosoureas such as fotemustine and carmustine (47). Platinum analogs (e.g., cisplatin) and antimicrotubular agents such as vinblastine and paclitaxel have also shown modest efficacies in patients with metastatic melanoma (47). Recently, clinical studies have been performed using biochemotherapy, which combines cytotoxic drugs with immunotherapies such as interleukin-2 (IL-2) and IFNα (interferon alpha), and despite showing increased response rates these patients did not experience prolonged overall survival (OS) (18). In patients with recurrent metastatic melanoma in the limb, high doses of the cytotoxic drug melphalan and recently, tumor necrosis factor (TNF) and IFNγ are given to the patient via isolated limb perfusion to reduce systemic toxicity (48). A significant improvement in melanoma treatment was observed using targeted therapies, which pharmacologically inhibit key mutations in melanoma. These include the BRAFi drugs vemurafenib and dabrafenib, and the MEK inhibitor trametinib (39). Targeted therapies for melanoma have been expertly reviewed elsewhere (39, 49). The major clinically approved immunotherapies for melanoma include adjuvant treatments such as IL-2 and interferon alfa (18, 48). A few clinical groups have had success with adoptive T cell therapy in a subset of patients (50). Finally, immune checkpoint blockade (ICB) with antibodies targeted to cytotoxic T lymphocyte antigen-4 (CTLA-4) (ipilimumab) and programmed cell death protein 1 (PD-1) (nivolumab and pembrolizumab) has resulted in significant improvements in clinical outcomes for a proportion of melanoma patients (39). Targeting the ligand for PD-1 (i.e. PD-L1) is also being studied in clinical trials (51, 52). This review will summarize the evolution of immunotherapies in the context of melanoma and discuss novel opportunities to significantly enhance tumor immunotherapy. To assess the results of clinical studies, it is pertinent to mention some of the key measures used in clinical trials and criteria defined within the RECIST (Response Evaluation Criteria in Solid Tumors) (53). OS is defined as the time from randomization of the treatment subject to time of death due to any cause, while the more utilized progression-free survival (PFS) metric, denotes time from randomization until tumor progression or death (54). The overall objective response rate (ORR) is a measure of the percentage of patients who have had either a partial response (PR) or complete response (CR) to treatment (54). PR is defined as a decrease of at least 30% in the sum of the diameters of the target tumor lesions while CR indicates the disappearance of all target lesions (53). Finally, progressive disease (PD) is defined as at least a 20% increase in the sum of the target lesions’ diameters while stable disease (SD) denotes a state where the lesions do not shrink enough to signal PR or increase sufficiently to indicate PD (53). Thus, these parameters provide an objective methodology to measure the results of a treatment (53, 54).

## Immunobiology of Melanoma

### Cancer Immunoediting

Over the past decade, cancer immunotherapy has emerged as a vital new approach to cancer treatment (55, 56). The earliest evidence of the involvement of the immune response in fighting cancer was observed over a century ago. In 1893, William Coley, a surgeon in New York published a report describing tumor regression in a number of patients treated with cultures of the bacterium *Streptococcus pyogenes* (57, 58). However, the immunological basis of these results was not yet known and the approach did not gain wide acceptance in the medical field. Nevertheless, subsequent observations in murine models led to the formulation of the “cancer immunosurveillance” hypothesis by Macfarlane Burnet and Lewis Thomas in the middle of the century (59, 60). The hypothesis posited that lymphocytes played a protective role by continuous recognition and elimination of malignant cells (61). Currently, the concept of “cancer immunoediting” is forwarded as a comprehensive depiction of the continuous interplay between tumors and the immune system (62, 63). Cancer immunoediting posits the existence of three distinct phases, namely, elimination, equilibrium, and escape (63, 64). In the *elimination* phase, innate and adaptive immune mechanisms eradicate neoplastic cells before they become clinically detectable cancers (64). This phase has not been directly observed *in vivo* but the increased susceptibility to developing cancer in immunodeficient mouse models provides evidence of the existence of this stage of immunoediting (64). Further observations in humans such as the increased risks of cancers in patients with immunodeficiencies or undergoing immunosuppression for organ transplantation, as well as cases of spontaneous tumor regression lend further proof to this paradigm (64, 65). During the *equilibrium* stage, rare cancerous cells that were not destroyed during the elimination phase, are kept in check by the immune system while influencing the immunogenicity of the tumor (62). This state results in a form of tumor dormancy and is considered to last a long time, potentially lasting the lifetime of an individual. Furthermore, this phase enacts a selective pressure on the tumor cells, allowing those with the potential to evade the immune system to escape immune control and manifest as clinical disease (62, 64). A landmark study in 2007 demonstrated the existence of the equilibrium phase *in vivo*. Using a carcinogenic compound (3′-methylcholanthrene -MCA), the authors were able to study stable tumor masses at the site of MCA injection (66). When treated with a cocktail of antibodies targeting CD4, CD8, and IFNγ, 60% of the mice developed rapidly growing tumors. Furthermore, the authors demonstrated that these rapidly growing tumors resembled “unedited” tumors from MCA-injected RAG^−/−^ mice (mice lacking recombination activation gene RAG1) (66). Finally, it was shown that this equilibrium state required components of adaptive immunity (IL-12, IFNγ, CD4^+^, and CD8^+^ cells) but not key components of innate immunity such as NK cell recognition and effector functions (66). Thus, while the immune system is capable of controlling cancerous cells during the equilibrium phase, it also drives the selection of cells that are able to evade immune attack and develop into a progressively growing tumor. This stage is known as the *escape* phase of immunoediting. This escape is made possible due to a number of potential mechanisms which have been reviewed in detail (61, 63, 65). Briefly, the cells can evade immune detection by reducing the expression of immunogenic tumor antigens or by reducing major histocompatibility complex class I (MHC I) (62, 64). Another route of escape involves decreased susceptibility to immune-mediated cytotoxicity through upregulation of oncogenes and anti-apoptotic mediators (64). Finally, tumor cells harbor the potential to modulate the immune system by producing immunosuppressive cytokines such as transforming growth factor beta (TGFβ) and vascular endothelial growth factor (VEGF). Moreover, tumor cells can recruit regulatory immune cells [e.g., regulatory T cells (Treg)] or engage in adaptive immune resistance via the expression of immune checkpoint ligands such as programmed death-ligand 1 (PD-L1) (64). Finally, the notion of “reverse immunoediting” has been proposed as some cancers can cause the selective depletion of specific high-avidity cytotoxic T cell (CTL) clones via hitherto unknown mechanisms and thus actively shape the immune repertoire of the host (67). The pathways used by tumor cells to escape the immune system are therefore studied extensively to devise immunotherapeutic approaches for cancer treatment.

### Immune Response to Melanoma

**Figure 2:**
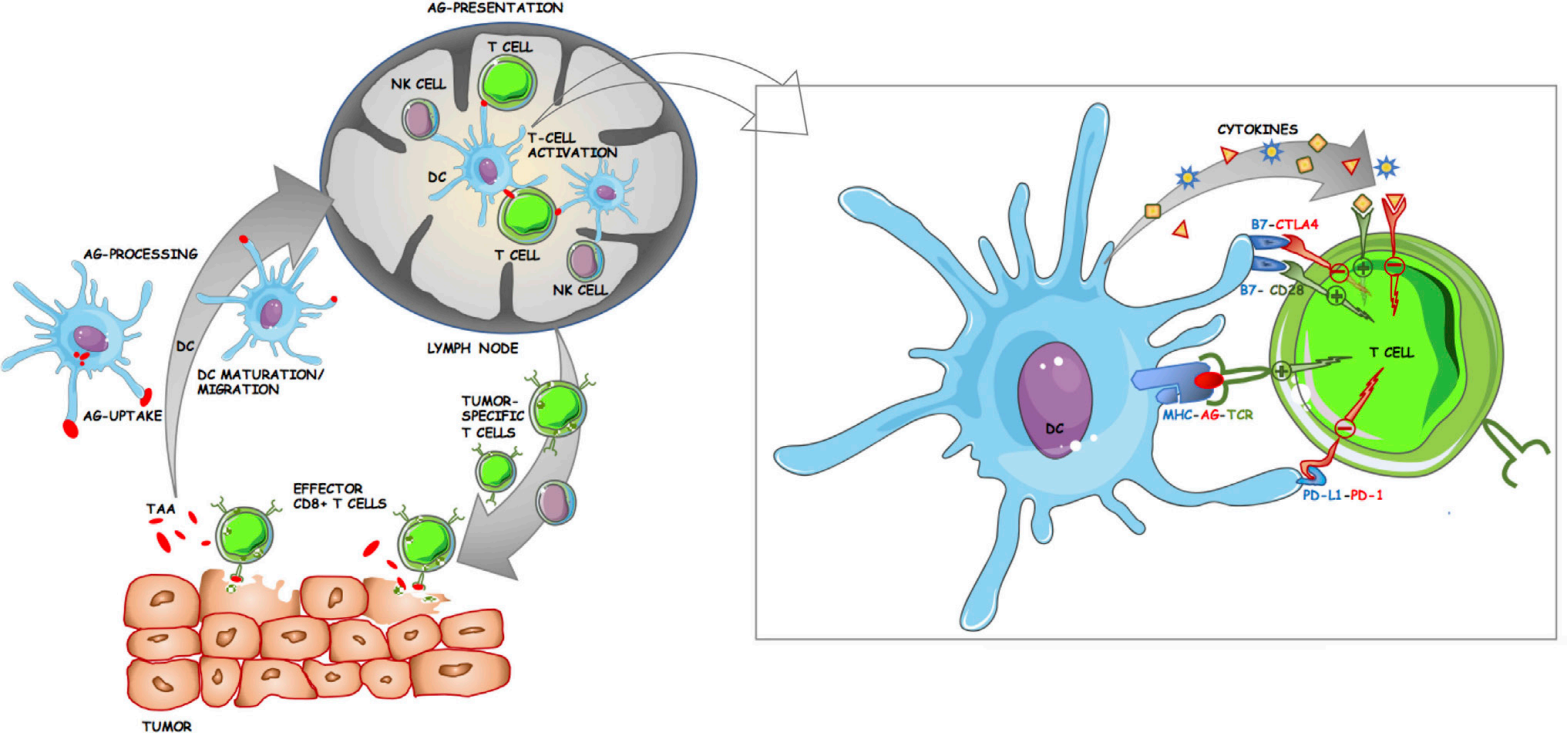
Schematic of the roles dendritic cells (DC) play in antitumor immune response. DC take up and process tumor-associated/tumor-specific antigens (TAA/TSA) from dying tumor cells, undergo maturation, and migrate to tumor draining lymph nodes (LN) where they can present antigen to lymphocytes. Tumor-specific T cells then egress from the LN and infiltrate the tumor. Effector CD8+ cytotoxic T lymphocytes play a major role in killing tumor cells, leading to further release of TAA/TSA for DC uptake and subsequent presentation. Inset panel: Costimulatory and inhibitory interactions at the antigen-presenting cell (APC)–T cell immunological synapse. The activation of T cells by APC is tightly regulated by multiple ligand–receptor interactions. TCR binds to cognate antigen (AG) in the context of their specific MHC. Costimulatory molecules such as CD80 (B7.1) and CD86 (B7.2) on APC can either bind to CD28 on T cells resulting in downstream activation of T cell effector genes or to cytotoxic T lymphocyte antigen-4 (CTLA-4) resulting in inhibition. Further T cell activation is achieved through cytokines. Programmed cell death protein 1 (PD-1) is another immune checkpoint receptor and is expressed on activated T cells. The primary ligand for PD-1 (PD-L1) is expressed on APC and on some tumor cells, and upon binding to PD-1 acts to inhibit T cell activation.

The immune response to tumor cells is currently one of the major areas of research in biomedical science. An overview of antitumor immune response is provided by the concept of the cancer-immunity cycle as described by Chen and Mellman (68). It commences with the release of tumor antigens that are presented by antigen-presenting cells (APC), primarily dendritic cells (DC), to T cells in the LN (Figure 2). This is followed by the trafficking of T cells including CD8^+^ cytotoxic T lymphocytes (CTL), to the tumor where they can recognize and kill malignant cells, thereby releasing more cancer antigens (68). However, at each step, there are negative regulators that can disrupt the cancer-immunity cycle and allow progression of the tumor (68). One of the primary aims of cancer immunotherapy is therefore to ensure a sustained T cell response against the tumor (55). The complex biology of the interactions between tumor cells and the innate and adaptive immune system has been extensively reviewed elsewhere (68–72). Thus, the primary focus of this section will be to provide a basic primer to cancer immunology and in particular, to the biological and therapeutic significance of the major types of immune cells in the tumor microenvironment (TME) in melanomas. For the purposes of this review, the populations of interest are tumor-infiltrating lymphocytes (TIL), tumor-infiltrating dendritic cells (TIDC), and tumor-infiltrating natural killer (NK) cells. The cancer-specific roles of tumor-associated macrophages (TAM), NKT cells, the more recently described myeloid-derived suppressor cells (MDSC), and non-NK innate lymphoid cell subsets (ILC) have been thoroughly reviewed elsewhere (73–77).

#### Tumor Antigens

As tumors arise from a host’s own tissue, immune recognition of these cells is hindered by the fact that a majority of potentially autoimmune cells are deleted during central (thymic) and peripheral mechanisms of self-tolerance (78). However, as early as 1943, it was observed that mice could immunologically reject chemically induced tumors (79). In the late 1970s, the ability to grow CTL cultures using IL-2 allowed for screening of tumor-derived DNA libraries to characterize tumor antigens (79). In 1988, the gene coding for a murine tumor antigen (P91A) was cloned (80). Shortly afterward, the first human tumor antigen gene was identified in melanoma, namely, *MAGEA1* (melanoma antigen family A, 1) and was found to be expressed in various types of tumors (81). Interestingly, the gene was not observed to be expressed in normal tissue except for trophoblastic cells and male germline cells (79). Since then, several tumor antigens have been discovered, and their underlying biology has been the subject of much study (82, 83). There are several types of tumor antigens, but they have been broadly classified into three major categories. The first category includes antigens that are caused by non-synonymous mutations, or are encoded by viral genes in tumors of viral etiology (83). These are labeled tumor-specific antigens (TSA) or “neoantigens” (83, 84). Alternately, tumor-associated antigens (TSA) are usually expressed at low levels in normal tissues but are found to be overexpressed in cancer cells such the surface receptor, human epidermal growth factor 2 (HER2 or ERBB2) in breast cancer, and other malignancies (85). Finally, cancer/testis antigens (CTA) such as the aforementioned MAGE family of proteins are expressed in several tumor types and only in normal germline cells such as trophoblasts, ovaries and the testes (82, 83). The advent of high-throughput next-generation sequencing technology has allowed for relatively low-cost detection of somatic mutations in tumor cells. There are currently several approaches being formulated to tailor individualized immunotherapies for patients on the basis of their expression of tumor neoantigens (83). Although currently personalized approaches are highly expensive, it is posited that with the continuing reduction of sequencing costs and using combinatorial treatments, it may be possible to even target tumors that are non-responsive to immunotherapy (83). Since their discovery, tumor antigens have been used for multiple purposes in cancer treatment. They have been used as diagnostic markers, cancer vaccines, and as targets for adoptive T cell therapy (82, 86, 87). In general, most tumor antigens elicit a weak immune response against cancer and have been tested clinically in combination with adjuvants or additional treatments (87). To date, cancer vaccination or adoptive transfer targeting specific tumor antigens has not shown major survival advantages in melanoma (48, 88). The three major types of tumor antigens that have been described and used in melanoma immunotherapy are discussed below. A majority of described melanoma antigens are restricted to human leukocyte antigen A2 (HLA-A2) (89).

##### MAGE Family

The MAGE (melanoma antigen) family is divided into two major groups type I MAGEs and type II MAGEs. The type I MAGE subfamily consists of 25 functional genes located on the X chromosome in the regions *MAGEA, MAGEB*, and *MAGEC* (82, 90). These genes are classified as CTAs and are expressed in melanoma as well as other cancer types such as colon cancer, non-small cell lung cancer (NSCLC), and breast cancers (90). Conversely, type II MAGE genes are expressed in several types of normal tissue and are not X chromosome restricted. Both type I and type II MAGEs contain the MAGE homology domain (90). Due to the extensive homology between the MAGE proteins, there is a lack of antibodies that recognize specific MAGE antigens. In several cancer types, nuclear and cytoplasmic staining using widely reactive anti-MAGE antibodies have been performed and although the functions of MAGE proteins are not known, there is some evidence that they play a role in cell cycle progression and apoptosis (91). The MAGE family of proteins may serve as useful targets for immunotherapy. After encouraging results from Phase I/II studies, the DERMA phase III clinical trial aimed to assess a vaccine using MAGE-A3 protein in combination with an immunostimulant, in melanoma patients following tumor resection (92). However, in 2016 the trial was ended as it failed to show efficacy (NCT 00796445). Nevertheless, the lack of MAGE family gene expression in normal tissue and their overexpression in cancer cells is one of the key reasons they remain attractive targets for future immunotherapy treatments. Other CTAs observed in melanoma include the B-M antigen-1 (BAGE) and G antigen (GAGE) family of proteins, and their functions are currently being studied (86).

##### NY-ESO-1

NY-ESO-1 (New York esophageal squamous cell carcinoma-1) is a CTA that is also located on chromosome X and is expressed in a wide range of malignancies (93). In normal cells, this antigen is primarily expressed on spermatogonia and at very low levels in pancreas, liver, and placenta (93). A homolog of NY-ESO-1, LAGE-1 has also been reported and is expressed in a wide variety of human cancer types. The biological functions of both proteins are unknown (93). NY-ESO-1 is a highly immunogenic tumor antigen and is able to elicit a detectable antibody response. In human melanoma, it is observed in a large frequency of melanoma patients (46%) and some studies indicate that its expression may be higher in metastatic lesions (93, 94). Due to its expression in a large fraction of melanomas, immunotherapy trials continue to be conducted using the NY-ESO-1 antigen as part of a tumor vaccine, or more recently using adoptively transferred lymphocytes with recombinant TCRs specific for NY-ESO-1 (95, 96). The adoptive transfer trial resulted in objective responses in 55% of treated melanoma patients but the most efficacious strategy for targeting NY-ESO-1 in melanoma immunotherapy remains to be determined.

##### Melanoma Differentiation Antigens

A number of TAA in melanoma that are recognized by both CD4^+^ and CD8^+^ T lymphocytes are on proteins specifically expressed on melanocytes and involved in melanocyte-specific functions (86, 97). These TAA are located in melanosomes, the organelles in which melanin is synthesized. Moreover, their role in oncogenesis is not known (86). These antigens include *tyrosinase, tyrosinase-related proteins 1 and 2 (TRP-1 and TRP-2), Melan-A (MART-1)*, and *gp100 (pmel17)* (82, 97). *Tyrosinase* and *TRP-1/-2* are copper and zinc containing metalloenzymes with homology at several sequences and they play crucial roles in melanin synthesis (98). *Tyrosinase* is the key enzyme in melanin synthesis and is located on the membrane of melanosomes. It is observed in over 80% of primary and metastatic melanomas (86). The exact function of *TRP-1* (gp75) remains unclear, but it is purported to play a role in stabilizing tyrosinase (98). *TRP-2* is a DOPAchrome tautomerase and its overexpression is believed to contribute to the chemoresistance and radiotherapy resistance of metastatic melanoma (86, 97). *Melan-A* (melanoma antigen recognized by T cells-1 or MART-1) is a single domain transmembrane protein of 118 amino acids found primarily in melanosomes, endoplasmic reticulum, and trans-Golgi network (86, 99). MART-1 is crucial for the expression, trafficking, and stability of the protein gp100 (pmel17) (99). It is expressed in all melanocytic naevi, and a majority of primary and metastatic melanomas (86). It has been observed that significantly higher frequencies (100- to 1,000-fold) of naive CTL are found against a specific MART-1 peptide (Melan-A_26–35_) compared to other antigens in normal (non-cancerous) individuals who express HLA-A2 (79). However, T cell recognition of MART-1 does not necessarily result in improved clinical outcomes (97). Finally, the protein gp100 (premelanosomal protein-pmel17), is a transmembrane protein that has a role in melanosome biogenesis and melanin polymerization (86). The gp100 gene was found to be widely expressed in malignant melanoma at all stages but was significantly reduced in normal melanocytes (100). HMB-45, a mouse monoclonal antibody (mAb) to gp100, is used for diagnostic purposes to distinguish non-melanocytic from melanocytic tumors (99). All of the aforementioned differentiation antigens are recognized by CD4^+^ and CD8^+^ T cells, while TRP-1, TRP-2, tyrosinase, and gp100 can also elicit antibody responses (97). Thus, these antigens are considered to be useful targets for melanoma immunotherapy (86). The B16 syngeneic transplant model, obtained initially from C57BL/6 mice, is one of the most widely utilized models in melanoma research (101). The most obvious advantage of this model is that it expresses murine homologs of the melanoma differentiation antigens (tyrosinase, gp100, MART-1, TRP-1, and TRP-2) (102). Melanocyte differentiation antigens continue to be used in a number of clinical studies in combination with various adjuvants and immunostimulants such as granulocyte-macrophage colony-stimulating factor (GM-CSF), but none of the studies have to date shown significant improvements in OS in melanoma patients (87, 103, 104). Due to the multiple mechanisms of tumor immune escape, it remains particularly difficult to sustain a prolonged response to cancer antigens. However, recently the use of nanoparticles (NP) containing mRNA encoding the melanoma antigens, NY-ESO-1, tyrosinase, MAGE-A3, and a novel CTA TPTE (a transmembrane phosphatase), has shown early clinical promise in a pilot study of three patients (105). To be successful, future immunotherapy trials will need to not only consider the tumor antigens to be used but also the delivery vector, the format (RNA, DNA or protein), and the appropriate adjuvants.

#### Tumor-Infiltrating Lymphocytes

A cardinal feature of cancer is the immunosuppressive TME (106, 107). As the disease progresses, T cells in the TME exhibit a phenotype analogous to that seen in chronic viral infection known as T cell exhaustion (108). T cell exhaustion denotes a state of hyporesponsiveness to antigen with reduced cytokine secretion and cytotoxic function (108, 109). Nevertheless, the overwhelming majority of studies in human patients have demonstrated a correlation between TIL and better disease outcomes in cancers (110, 111). An exception to this observation is that FOXP3 expression, a marker of Treg that has been shown to correlate to poor prognosis in various types of human cancer (112, 113). The term TIL was first described by Wallace Clark, who was instrumental in developing the first histological classifications for melanoma as mentioned above (114, 115). TIL have been described in primary tumors, tumor-bearing LN, and in metastases of melanoma and various other cancer types (114). The range of immune cells that infiltrate a tumor, i.e., the “immune contexture” of a tumor is heterogeneous and consists of various types of T lymphocytes, B cells, NK cells, macrophages, and DC (111, 114). In 1989, Clark published a classification of the three major patterns of lymphocyte infiltration that are commonly used today (115). The *brisk* pattern is indicated by interposed lymphocytes between tumor cells that may be diffusely present throughout the tumor nodule or along the advancing (basal) periphery of the nodule (114, 115). The *non-brisk* pattern delineates a scattered multifocal presence of lymphocytes throughout the vertical growth phase of the nodule. Finally, an *absent* pattern is associated with a lack of lymphocytes in the tumor, or if they are present, their lack of interaction with melanoma cells (115). In recent years, various groups have attempted to further classify TIL or propose novel grading schemes, but the Clark model remains widely accepted and highly reproducible (114). In a recently published report, it was shown that melanoma tumors with *brisk* TIL patterns in primary melanoma H&E tissue, even in the absence of immunohistochemistry for specific markers, was associated with increased OS in patients versus tumors with *non-brisk* and *absent* patterns (116). The importance of TIL has been used to establish a novel classification system for cancer based on an “Immunoscore,” which relies upon the quantitation of CD3 and CD8 lymphocytes with the additional marker CD45RO used to mark memory T cells. The “Immunoscore” was found to be superior to the conventional AJCC TNM system for prognosis of stage I–III colorectal cancer (CRC) (117). Similar approaches are now being tested for immunoscoring of melanoma but have not been tested in large patient cohorts (118).

An additional feature observed in cancer, and other situations of chronic inflammation is the formation of tertiary lymphoid structures (TLS—also called tertiary lymphoid organs) (119, 120). These TLS can range from loose aggregates of various immune cells to complex structures that resemble secondary lymphoid organs such as LN. They consist of T cell-rich regions containing mature DC expressing DC-LAMP (lysosomal associated membrane protein), B cells, and high endothelial venules, which play a role in immune cell extravasation and production of key chemokines (120). In 2012, Messina et al. reported that a gene expression profile consisting of 12 chemokines could accurately predict the histological presence of LN-like TLS in stage IV melanoma (primary tumors and metastases), and the TLS correlated strongly with improved overall patient survival (121). Other studies have shown that the presence of TLS is a positive prognostic indicator in melanoma and a range of other cancer types including breast carcinoma, CRC, and pancreatic cancer (120). Thus, these results suggest that lymphocyte infiltration mediates a protective immune response to cancer.

However, many tumors are not T cell inflamed, and the mechanisms underlying T cell infiltration into the tumor are poorly understood (89, 122). In the context of melanoma, a recent study compared all major classes of melanoma tumor antigens between T cell inflamed and non-T cell inflamed tumors and found that there were no differences between both groups in terms of antigen load (123). Rather it was shown that non-T cell inflamed melanomas displayed reduced gene expression associated with Batf3-dependent, CD141^+^ DC (123). Furthermore, studies have pointed to the ability of tumors to interfere with chemokines that recruit leukocytes to tumors. Finally, the abnormal tumor vasculature may express reduced adhesion molecules required for homing and directly or indirectly suppress T cells by expression of molecules such as PD-L1, PD-L2, VEGF, and TGFβ (122). Once T cells infiltrate the TME, they are acted upon by a range of immunoregulatory mechanisms that prevent complete eradication of the tumor (72). These can be tumor-specific escape mechanisms or the recruitment of suppressive immune cells. For instance, mutations in BRAF or PTEN loss are associated with increased T cell inhibition by production of IL-1 and VEGF (72). Furthermore, conserved immunoregulatory mechanisms are also at play within the TME the production of immunosuppressive mediators [TGFβ and indoleamine 2,3 dioxygenase (IDO)], and the recruitment of regulatory myeloid and lymphoid cell populations (72). Another important consideration is that although, CD8^+^ T cells are canonically considered the primary cytotoxic cells involved in tumor eradication, CD4^+^ T cells can also kill tumor cells (89). However, the precise mechanisms of CD4^+^ antitumor immunity are not well described, and the role of CD4^+^ T cell infiltration in the TME has not been explored significantly with the exception of FOXP3^+^CD4^+^ Treg (72, 89). A recently concluded meta-analysis demonstrated that FOXP3^+^ Treg infiltrates were predominantly associated with worse OS in a review of over 17 types of cancer (124). In most tumors, such as cervical, renal, breast cancers, and melanoma, FOXP3^+^ Treg infiltrates correlated with shorter OS whereas they were associated with improved survival in patients with colorectal, head and neck, and esophageal cancers (124). In recent years, several studies have described the heterogeneity in FOXP3-expressing cell populations (125). In 2016, Saito et al. showed that human CRCs could be distinguished by the extent of infiltration of two distinct FOXP3^+^CD4^+^ T cell populations (126). Type A CRCs had low frequencies (<9.8%) while Type B had comparatively higher frequencies (>9.8%) of infiltrating non-suppressive FOXP3^lo^CD45^−^ T cells. Infiltration by these non-suppressive T cells was correlated with the presence of intestinal bacteria, in particular *Fusobacterium nucleatum* within the tumor (126). Furthermore, Type B CRCs were marked by high mRNA expression of *IL12A* and *TGFB1* compared with Type A and tumors with high expression of these mRNAs exhibited significantly longer disease-free survival versus low expressing tumors. Thus, FOXP3^+^ T cell infiltration must be considered in combination with other immune signatures while determining the immune status of a tumor. In addition to T cells, the roles of B cells in the TME are being currently explored as they have both APC and effector lymphocyte functions (127). Studies in melanoma have demonstrated that CD20^+^ infiltrating B cells are found in most tumors and higher levels of these infiltrates correlated with improved patient survival (127). Furthermore, B cells are known to produce IgG antibodies that can recognize tumor cells and within a murine model of organ transplantation have been observed to promote chronic allograft rejection through antigen presentation rather than their antibody secreting functions (127, 128). Finally, recent studies have also focused on the roles of putative regulatory B cells in the context of transplantation and autoimmunity, as these cells can produce potent immunosuppressive mediators such as IL-10 and TGFβ (129). The multiple immunoregulatory mechanisms that effect TIL are the targets of a majority of current immunotherapies. However, as the aforementioned observations indicate, there are several functionally redundant pathways that allow for immunological escape of tumors in immunocompetent individuals. Thus, to be successful, the field of immunotherapy must move toward combinatorial and multipronged approaches for tumor treatment. This involves investigation of the mechanisms of innate immune cells such as NK cells, TAM, and TIDC within the TME.

#### Tumor-Infiltrating Dendritic Cells

Despite their discovery over 40 years ago, the exact mechanisms underlying DC dysfunction in cancer remain poorly understood (107). In both mice and humans, DC are classified into two major subsets comprised of conventional or cDC, and plasmacytoid DC (pDC) (130). In non-steady state conditions such as cancer or autoimmune disease, inflammatory DC derived from monocytes have also been described in humans and in mice (130, 131). Despite the fact that nearly all DC subsets express the surface marker CD11c, there are unique transcription factors and surface proteins that characterize the major DC subsets in human and mice. These markers have been extensively reviewed in the literature, but further study is needed to accurately profile each subset (130, 132, 133). DC canonically present extracellular antigens on MHC class II while intracellular or self-antigens are presented on MHC class I (134). However, murine and human DC also possess the capacity to cross-present antigens of extracellular origin on MHC class I to activate CD8^+^ CTL (135, 136). In humans, the primary cross-presenting DC subset is characterized by CD141 (BDCA-3) while in mice this subset is marked by surface expression of CD8α or CD103 (137). The mechanistic roles played by various DC subsets in both tumor progression and the response to treatment are a key area of research for cancer immunotherapy with little consensus as to their frequencies and functions (102, 107). In 2008, it was reported that knocking out *Batf3* in mice eliminated CD8α^+^ DC, and consequently it was demonstrated that these mice were incapable of cross-presenting antigen or rejecting highly immunogenic fibrosarcomas (138). Although pDC are purportedly not efficient at cross-presentation, studies have shown their capacity to mediate direct tumor killing and to activate NK cells via the production of type I IFN (139). Despite the key roles played by TIDC in promoting antitumor responses, generally TIDC are skewed in both phenotype and function toward an immunosuppressive role in the microenvironment (107). These alterations in TIDC have been mechanistically studied in murine models (107, 140). The TME has been reported to induce a “paralyzed” state in TIDC resembling an immature phenotype with reduced expression of costimulatory CD80 and CD86 molecules and a diminished capacity to present antigens (107). This induction is a result of various immunosuppressive factors such as VEGF, TGFβ, IDO produced by tumor cells as well as by other cells in the TME (72, 107). Furthermore, DC paralysis in mouse models has been observed to be associated with upregulation of immune checkpoint receptors such as PD-1 and T cell immunoglobulin and mucin-domain containing-3 (TIM-3), which was reported to interact with the alarmin protein high mobility group box 1 (HMGB1) resulting in reduced DC sensing of tumor-derived nucleic acids (107). TIDC with immature and paralyzed phenotypes themselves suppress immune cells in the TME through various mechanisms such as but not limited to, expression of inhibitory molecules (PD-L1), production of regulatory cytokines such as IDO and induction of Tregs (107, 141).

As previously noted, there has been significant research on TIL in melanoma. On the other hand, the mechanistic roles of TIDC in melanoma are not well studied. Melanoma is of particular interest due to the fact that skin contains multiple DC subsets. The five major DC subsets found in human skin are Langerhans cells, CD14^+^ DC, CD1c^+^ DC, CD1a^+^ DC, and CD141^+^ DC (133). The correlations between various TIDC subsets and disease outcome, their association with other cells and specific functions have not yet been fully elucidated (102). However, recently it was demonstrated that intratumoral CD103^+^ DC in mice were crucial for trafficking of melanoma tumor antigen to LN and were dependent on surface expression of CCR7 (142). Enhanced CCR7 mRNA expression in human melanoma samples was also correlated to increased T cell infiltrates and improved patient outcomes (142). In general, it is observed that there are higher frequencies of TIDC in the peritumoral region than within the tumor (102). These peritumoral DC include arguably the most mature population of DC-LAMP^+^CD83^+^fascin^+^ cells (102). In fact, DC-LAMP expression is associated with positive prognosis in not only melanoma but also lung, breast, and metastatic CRC (120). On the other hand, CD123^+^ pDC that do in principle possess the capacity to promote antitumor responses are found to be associated with early relapse and poor prognosis in human melanoma (102, 143). It was shown in both *ex vivo* patient samples and in that a humanized melanoma mouse model that pDC in melanoma are directed toward a T_H_2 promoting phenotype by induction of the molecules OX-40L (TNFSF4) and ICOSL (inducible T cell costimulator ligand), which then drive tumor progression (143). To comprehensively characterize TIDC in melanoma, it is crucial to obtain genomic data to appropriately distinguish and profile TIDC subsets. Pyfferoen et al. performed transcriptomic profiling of DC in a murine model of lung carcinoma and demonstrated that TIDC had significantly increased expression of PD-L1, acquisition of TAM surface markers and a pro-metastatic microRNA signature (144). To date, similar studies have not been performed in human melanoma. There have been several studies in murine models that have demonstrated the therapeutic reprogramming of TIDC (107). Thus, manipulation of TIDC represents a hitherto unexplored target for future melanoma immunotherapies. Many of the same agents that have been shown to induce DC activation and maturation *in vitro* have been tested for direct targeting of DC *in vivo* (133, 145). For instance, direct administration of BCG has been utilized for the treatment of bladder cancer for over 30 years although its precise mechanisms of action *in vivo* are still under study (146). Direct modulation of DC *in vivo* using DC maturation agents and mAbs is a highly desirable goal in tumor immunotherapy. This is due to the excessive costs, safety considerations, and practical limitations of using cellular products (147). As such, the identification of both targetable DC receptors and maturation stimuli continues to be an active area of research interest. In particular, targeting antigen-coupled antibodies to DC C-type lectin receptors (CLRs) such as DEC205 (CD205), Clec9A, and DC-SIGN in murine and *in vitro* studies resulted in effective CD4^+^ and CD8^+^ T cell responses (145, 148). Additional receptors such as XCR1 (expressed entirely on CD141^+^ DC) are also being studied for their effects on DC function (133). Clinical trials for multiple cancer types are presently underway to investigate the efficacy of anti-DEC205 conjugated to the cancer–testis antigen NY-ESO-1, which is also used for melanoma immunotherapy (133, 149). Recently, a series of seminal papers have shown the importance of the cytosolic DNA sensor cyclic GMP-AMP (cGAMP) synthase (cGAS) in promoting antitumor immunity (150–152). DNA introduced to the cytosol as a result of viral infections or cellular damage is a potent immune activator that leads to the production of type I IFN (153). Upon detection of DNA by cGAS, it catalyzes the production of cGAMP that binds to the adaptor protein stimulator of interferon genes (STING) ultimately resulting in the production of type I IFN (153). In 2014, Woo et al. demonstrated in a mouse model that tumor-derived DNA was responsible for inducing IFNβ production and the consequent activation of APC and CD8^+^ T cells versus melanoma *in vivo* (150). Alternately, mice deficient in STING failed to reject these tumors highlighting the crucial role played by this pathway in the immune response to cancer (150, 151). In a more recent paper, Wang et al. showed the role of cGAMP in mediating the effects of ICB (152). It was reported that in mice lacking either cGAS or STING, PD-L1 blockade did not result in significant shrinkage of tumor volume or increase in survival compared with WT mice. Moreover, intramuscular injection of cGAMP in combination with PD-L1 significantly enhanced survival, compared with PD-L1 or cGAMP alone (152). Finally, it was also shown that cGAMP treatment of BMDC enhanced expression of DC activation markers and increased DC antigen cross-presentation. Another molecule that has recently gained interest for its effects on DC is IL-32. In 2012, Schenk et al., identified an IL-32-dependent mechanism for DC differentiation in response to nucleotide-binding oligomerization domain containing protein (NOD2) activation through its ligand muramyl dipeptide (154). DC obtained from IL-32 differentiation were found to express higher levels of MHC class I and CD86, as well as, present antigen to CD8^+^ T cells more effectively than GM-CSF differentiated DC (154). These studies highlight the multiple pathways that may be targeted to generate effective DC *in vivo*, which is essential for antitumor immunity.

#### NK Cells

Natural killer cells were characterized over 40 years and are the first population of ILC to be described and studied (155, 156). NK cell defects lead to enhanced susceptibility to viruses and many forms of cancer in humans and in mouse models (156). NK cell functions are modulated by a number of surface receptors that provide either NK activating or inhibitory signals (156, 157). NK cells are broadly defined as CD3^−^CD56^+^ in humans and CD3^−^NK1.1^+^ in mice while both murine and human NK cells express the surface receptor NKp46 (CD335) (156). In humans, NK cells are further divided into CD16^+^CD56^dim^ which predominate in blood, and CD16^−^CD56^bright^ populations (156). Canonically, NK cells can recognize tumor cells that have downregulated MHC class I molecules or upregulated induced stress molecules (155, 156). NK cells can also bind to antibodies bound to tumor antigens and mediate antibody-dependent cellular cytotoxicity (156). As with CD8^+^ CTL, NK cells mediate their cytotoxic functions through perforin and granzymes, as well as, by expressing death mediating ligands such as FasL (CD95L) and TRAIL (TNF-related apoptosis inducing ligand) (156). Activated NK cells also produce IFNγ, among other cytokines, which leads to recruitment of other immune cell populations (156).

The roles of NK cells in the TME are currently not fully described (155, 157). Several studies have indicated that NK cell infiltration is generally a positive prognostic factor in various types of cancer (155). In the context of melanoma, the roles of NK cells are an important venue of research. Analysis of several melanoma cell lines indicated that a high percentage of melanoma cells possess ligands for a NK activating receptors such as NKG2D and DNAM1, while ligands have also been identified for NK-bound NCR (natural cytotoxicity receptors) such as NKp30 (157). Melanoma cells are also known to have decreased MHC class I expression as a mechanism to escape CD8^+^ T cells, thus making them targets for NK cells (157). Despite these observations, melanoma immunoediting leads to tumor escape from NK cells via multiple mechanisms (157). Melanoma immunoediting by NK cells increases expression of MHC I, or downregulates NK ligands supported by the decreased expression of MICA reported in metastatic versus primary melanoma (157). IDO and prostaglandin E2 (PGE2) produced by melanoma cells act directly to inhibit NK cells while increased expression of ligands to regulatory receptors such as TIGIT modulate NK cell activity (157). In light of these observations, it will be important to identify NK populations that have persistent antitumor activity and characterize their phenotypes to better understand the mechanism involved in effective NK immunity. Recently, it was reported that tumor-bearing/infiltrated LN in melanoma patients contained twice as many NK cells as ipsilateral tumor-free LN (158). These tumor-infiltrated LN also contained a population of highly cytotoxic CD56^dim^KIR^+^CCR7^+^ NK cells that may have prognostic potential for melanoma (158). Conversely, melanoma, breast, and colon cancers were found to be infiltrated by CD56^bright^ NK subsets, which are similar to decidual NK cells during pregnancy thus implying a potentially regulatory role for this subset (159). NK cells remain an important target for immunotherapy. Along with T cells, NK cells were used early on for adoptive cell transfer therapy of melanoma in the 1980s and both autologous and allogeneic NK cell adoptive transfers are being studied in clinical trials (156, 157). Currently, two antibodies for the blockade of NK checkpoints are under clinical development, namely, lirilumab (anti-KIR-studied in combination with ipilimumab) and IPH2201 (anti-NKG2A) for various types of cancers including melanoma (157). However, further study of NK cells in the melanoma TME is required to understand the several mechanisms of immune escape from NK cells and CD8^+^ CTL and thus devise, rational combinatorial immunotherapies.

## Melanoma Immunotherapy

In 2013, the journal *Science* hailed cancer immunotherapy as the breakthrough of the year (56). This was in recognition of the promising clinical responses that can be achieved by directing the immune system to fight cancer. Despite highly encouraging advances, current immunotherapies only result in clinical benefit for a subset of patients (160, 161). Thus, there is a significant scientific effort to understand the tumor cell-intrinsic and extrinsic mechanisms of resistance to immunotherapy (162). The three major mechanisms of resistance to immunotherapies have been conceptualized as follows. *Primary* resistance denotes a clinical setting where the initial immunotherapy is unsuccessful. This can be due to *adaptive* resistance which defines a mechanism whereby there are initial antitumor immune responses but are inhibited by adaptation and immune escape of the tumor (162). Clinically, adaptive resistance may be seen as primary resistance, mixed responses or *acquired* resistance. Acquired resistance describes a clinical scenario where the tumor initially responded to immunotherapy but has eventually progressed and acquired resistance to the therapy (162). To overcome resistance to various forms of immunotherapy, it will be important to understand the mechanisms that allow tumor cells to escape immune attack. The clinical experience with melanoma immunotherapies has shown significant promise and there is increasing evidence that a multipronged approach may be required to ensure durable responses in a majority of patients. This section describes the major immunotherapies that have already been developed or are under clinical development for the treatment of metastatic melanoma (summarized in Table 1). Advances in immunotherapy for other types of cancers, as well as, the use of mAbs to specifically target tumors have been previously reviewed in detail (163–166).

**Table 1:** Key immunotherapeutics and their primary mechanisms of action.

Treatment	Clinically tested agents	Mechanism(s) of action	Reference
**Immune activating mAbs**			
αCTLA-4	Ipilimumab (Yervoy^®^)	– Blockade of T cell checkpoint receptor – Depletion of intratumoral Treg	(160, 167)
αPD-1	Nivolumab (Opdivo^®^), pembrolizumab (Keytruda^®^)	– Blockade of T cell checkpoint receptor	(167, 168)
αPD-L1	Atezolizumab, durvalumab, avelumab	– Blockade of inhibitory checkpoint ligand expressed on immune cells and tumor cells	(167, 169)
αCD137 (4-1BB)	Urelumab	– Agonist of T cell costimulatory receptor	(170)
αKIR	Lirilumab	– Blockade of NK cell inhibitory receptor	(157, 171)
αLAG-3	BMS986016	– Blockade of T cell surface inhibitory molecule	(167)

**Adoptive T cell therapy**			
TIL	*Ex vivo* expanded TIL	– Infusion of pool of antitumor T cells	(50, 172)
Engineered T cells	Transgenic TCR or CAR bearing T lymphocytes	– Infusion of engineered T cells specific for tumor antigens	(50, 173)

**Vaccines**			
Cell-based vaccines	Tumor cells or activated DC/APC	– Induction of tumor-specific adaptive immunity	(87, 174, 175)
Peptide vaccines	Various tumor antigen peptides/lysates + adjuvant	– Induction of tumor-specific adaptive immunity	(165, 176)
Oncolytic viral vaccines	Talimogene laherparepvec (T-VEC/Imlygic™)	– Viral induction of tumor cell lysis and adjuvant mediated host immune activation	(177, 178)

**Cytokines**			
Interleukin-2	Aldesleukin (Proleukin^®^)	– Activates and expands T cells	(179, 180)
Interferon alpha	Interferon alfa 2b (Intron^®^ A, Sylatron™)	– Activates multiple facets of immunity and has direct effects on tumor cells	(181, 182)

*An overview of current immunotherapy approaches and their proposed mechanisms of action as discussed in this review. Trade names are provided for drugs that have received clinical approval in melanoma. References provided for further description of each approach*.

*KIR, killer-cell immunoglobulin-like receptor; DC, dendritic cells; APC, antigen-presenting cell; TCR, T cell receptor; CAR, chimeric antigen receptor; TIL, tumor-infiltrating lymphocyte; NK, natural killer; Treg, regulatory T cells*.

### Early Advances in Melanoma Immunotherapy

As previously noted, the mechanistic basis for Coley’s observations remained unknown for some time and during this time, surgery, radiation treatment, and cytotoxic chemotherapy became the primary means of cancer treatment. However, in the context of melanoma, two major forms of immunotherapy witnessed encouraging breakthroughs starting in the 1980s and led to renewed interest in the entire field. These breakthroughs occurred in systemic cytokine therapy with IL-2 and adoptive cell transfer using TIL (183). In 1985, Rosenberg et al., demonstrated in C57BL/6 mice that intraperitoneal injections of recombinant IL-2 were capable of significantly attenuating pulmonary metastases from tumors generated by the MCA-105 and -106 syngeneic sarcoma and B16 syngeneic melanoma lines (184). Retrospective analyses of metastatic melanoma patients who had been treated with IL-2 demonstrated an ORR of 16% and represented a significant advance in the treatment (185). IL-2 received FDA approval in 1998 for metastatic melanoma. However, as systemic treatment of IL-2 resulted in various toxicities, several groups have shifted to intralesional administration of IL-2, which resulted in CR rates of between 41 and 76% in various trials (48). In parallel to the successes achieved with IL-2, Rosenberg and colleagues reported the first successful use of adoptive T cell transfer for the treatment of solid cancers (186). Patients were treated with IL-2 and autologous TIL expanded from surgically resected melanomas. Objective responses were observed in 60% (9/15) of treated patients (186). Subsequently, in 2002, this approach was combined with lymphodepletion prior T cell transfer and demonstrated enhanced responses in patients (50). Currently, adoptive cell therapy (ACT) using TIL remains one of the most effective therapies for metastatic melanoma (183).

### Immune Checkpoint Blockade

Drugs that mediate ICB by targeting the inhibitory receptors CTLA-4 and PD-1 (Figure 2 *inset panel*) have been shown to induce durable responses in subsets of patients with various types of cancer including melanoma, NSCLC, and renal cell cancer (RCC) (187–190). Furthermore, antibodies targeted to the PD-1 ligand, PD-L1, are undergoing clinical trials and have resulted in objective responses for multiple cancer types (51, 191). To date, the FDA has approved four mAbs for ICB therapy: (1) ipilimumab (αCTLA-4); (2) nivolumab (αPD-1); (3) pembrolizumab (αPD-1); and (4) atezolizumab (αPD-L1) (192). They have been approved for various advanced and metastatic cancers ranging from unresectable or metastatic melanoma to urothelial carcinoma (atezolizumab) (168, 192). Currently, only ipilimumab, nivolumab, and pembrolizumab have received FDA approval for melanoma (167). Due to the fact that checkpoint receptors play important roles in regulating autoimmunity, the major toxicities associated with the use of ICB drugs include a range of autoimmune symptoms labeled immune-related adverse events (IRAEs) (193). The incidence of IRAEs is quite high, ranging from 70% in patients treated with αPD-1/αPD-L1 antibodies to as high as 90% in patients treated with αCTLA-4 and require careful management in the clinic with immunosuppressive medications (193). As ICB results in objective responses for only a subset of patients, there is a crucial need to identify biomarkers that can potentially predict the efficacy of a particular ICB treatment or designate a particular subset of patients who may benefit from ICB therapy (194).

#### CTLA-4

Cytotoxic T lymphocyte antigen-4 (also termed cytotoxic T-lymphocyte-associated protein 4), is a crucial regulator of T cell activation and ipilimumab, a human IgG1 mAb targeted to this molecule was the first ICB drug to show clinical efficacy in advanced melanoma and a number of other cancer types (48, 195). CTLA-4 plays a key role in T cell immunity and its molecular biology has been recently reviewed elsewhere (167, 196). However, to understand the clinical role of CTLA-4 blockade, a brief summary of its mechanism of action is warranted. Naive T cells are modulated by APC through the interaction of multiple surface receptors in a region referred to as the “immunological synapse” (197). Canonically, naive T cells require 3 signals for complete activation (Figure 2 *inset panel*) (198). The engagement of the TCR by peptide antigen presented in the context of MHC, provides the first signal of T cell activation (signal 1) (198, 199). T cells require further signaling from the binding of costimulatory molecules on T cells such as CD28, to its respective ligands CD80/86 on APC (signal 2). Finally, the complete activation requires cytokines (IL-2) binding to their cognate receptors on T cells (Signal 3) (199). As an evolutionary checkpoint to autoimmunity, activated T cells induce surface CTLA-4 expression, which binds with greater affinity to CD80/86 and mediates T cell inhibition and cell cycle arrest (195, 200). CTLA-4 is also expressed constitutively on Treg (167). The crucial role of CTLA-4 in maintaining tolerance is demonstrated by the severe multiorgan autoimmune pathologies and early mortality (3–4 weeks) observed in CTLA-4^−/−^ mice (201). Humans with heterozygous germline mutations in CTLA-4 also exhibit autoantibodies, increased intra-organ lymphocyte infiltration and other symptoms of immune dysregulation (167).

In 2010, Hodi et al. demonstrated the clinical efficacy of ipilimumab in patients with stage III and IV unresectable and metastatic melanoma whose tumors were refractory to prior treatments (187). The treatment subjects received ipilimumab alone, ipilimumab plus the peptide gp100 or gp100 alone. Patients receiving ipilimumab alone or ipilimumab plus gp100 had significantly increased median OS compared with those receiving gp100 alone (roughly 10 versus 6 months) (187). Currently, ipilimumab has only received FDA approval for melanoma. However, a number of studies have shown modest responses to ipilimumab in other tumor types such as metastatic RCC and NSCLC, and it continues to be studied in clinical trials as combination therapy with PD-1/PD-L1 (discussed below) (160, 167). As mentioned previously, a number of immunological toxicities (IRAEs) are commonly observed to occur in patients treated with ipilimumab primarily in the skin, GI tract, and the endocrine system and in some rare cases result in deaths (193). The frequency of severe toxicities (grade 3 or 4) in the preliminary phase III trials of ipilimumab was demonstrated to be 20%, but this value was not significantly higher than the toxicities associated with many chemotherapy or targeted therapy drugs (163, 195). Most IRAEs can be resolved within 6–12 weeks of steroid therapy but for steroid-resistant adverse events, patients can also be treated with immunosuppressive antimetabolite drugs such as azathioprine and mycophenolate mofetil (193). Novel CTLA-4 blockade agents including modified versions of ipilimumab are also currently under study for a number of advanced solid tumors with the aim of improving safety profiles and tumor-specific delivery (202).

#### PD-1/PD-1 Ligand (PD-L1)

The most clinically successful agents for ICB to date target the inhibitory PD-1/PD-L1 axis (169, 195). The transmembrane receptor PD-1 (CD279) plays a crucial role in regulating antigen-specific T cell responses (169, 203). PD-1 is not only expressed on activated effector T cells but also on NK cells, B cells, macrophages, and Tregs (167, 203). Similar to the activating co-receptor CD28, PD-1 is acted upon by two distinct ligands PD-L1 (B7-H1, CD274) and PD-L2 (B7-DC, CD273) (203). Whereas PD-L2 expression has hitherto been observed only on professional APC (including B cells), PD-L1 is expressed on various tissue types such as epithelial tissue, vascular endothelium, stromal cells as well as tumor cells and virus-infected cells (167, 203). The induction of PD-L1 expression is generally in response to pro-inflammatory cytokines such as interferons, TNF-α, and VEGF (167, 169). PD-1 does not, as its name implies, directly induce cell death. The binding of PD-1 to its ligands instead serves to attenuate T cell activation by recruiting the tyrosine phosphatase SHP-2, which interferes with signaling downstream of the TCR and leading to decreased T cell growth and reduced cytokine production (203). However, PD-1 signaling can also reduce the expression of antiapoptotic genes while upregulating proapoptotic gene expression thus impairing T cell survival (167).

PD-1-deficient mice do not display as severe a phenotype as CTLA-4^−/−^ mice, developing glomerulonephritis and arthritis in a C57BL/6 background and autoantibody induced dilated cardiomyopathy in BALB/c mice as they age (204, 205). This is arguably due to the more direct inhibitory and Treg-related functions of CTLA-4, whereas PD-1 serves to limit T cell activation indirectly and prevent peripheral autoimmunity (169). As noted previously, in certain conditions of persistent antigen exposure such as in chronic viral infections or in cancer, T cells are observed to develop a dysfunctional or “exhausted” phenotype (72, 167). Such T cells are also marked by elevated expression of PD-1 and other inhibitory receptors such as TIM-3 and LAG3 (72). Furthermore, PD-L1 and/or PD-L2 are both observed to be expressed on a number of tumor-infiltrating APC and tumor cells themselves, not only as a result of cytokines but also due to alternative factors such as gain of chromosomes carrying PD-L1 and PD-L2 or the signaling of the epidermal growth factor pathway (167). Recent studies have shown that APC and tumor cells bearing PD-L1 play additive non-redundant roles in the suppression of antitumor immunity (206). Thus, blockade of the PD-1/PD-L1 axis remains a critical area of interest in tumor immunotherapy with studies on its efficacy in nearly 20 types of solid tumors and hematological cancers (169).

In the context of melanoma, nivolumab, and pembrolizumab, both of which target PD-1 have been shown to have significant clinical efficacies (160, 169, 195). In 2012, results from a phase I study comparing various doses of nivolumab in NSCLC, prostate cancer, CRC, renal cell carcinoma, and melanoma patients were reported (188). The highest activity was demonstrated in melanoma patients where the cumulative response rate (for all doses) was 28% compared with 27% for renal carcinoma and 18% for NSCLC (188). In the same year, an αPD-L1 antibody (BMS-963559) was tested in advanced cancers ranging from melanoma to RCC and was shown to have comparatively low response rates (6–17%) (191). A number of recently concluded trials have also demonstrated the potency of pembrolizumab. The large multicenter phase II trial KEYNOTE-002 examined the efficacy and safety of pembrolizumab in patients who had progressed on ipilimumab therapy, and in patients with BRAF mutations, those who had received either BRAF or MEK inhibitor treatment (207). Patients received either two separate doses of pembrolizumab (2 or 10 mg/kg) or chemotherapy of the investigators choice (carboplatin, dacarbazine, paclitaxel, and temozolomide). The results were highly encouraging as the 6-month PFS was shown to be 38% (10 mg/kg) and 34% (2 mg/kg) in the pembrolizumab group compared with only 16% in the chemotherapy group (207). Similar efficacy over investigator choice chemotherapy (32 versus 11%) has also been reported from an open-label phase III trial of nivolumab in patients who had progressed on ipilimumab (195). Furthermore, pembrolizumab was shown to have significantly higher activity than ipilimumab in patients with advanced melanoma. Robert et al. compared two dosing schedules (every 2 or 3 weeks) of pembrolizumab to ipilimumab and reported 6-month PFS in the range of 46–47% (response rates of roughly 33%) for the pembrolizumab group versus 26.5% (RR of 11.9%) for the ipilimumab-treated patients (208). Finally, in a phase III trial of nivolumab in previously untreated advanced melanoma patients (without BRAF mutations), ICB therapy was demonstrated to have significantly higher efficacy compared with dacarbazine with a 1 year survival rate of 72.9% in the nivolumab treated group versus 42% in the dacarbazine group (189). The successes of αPD-1 in melanoma treatment have also been observed (albeit at lower rates) in a range of other cancer types (167, 169). Furthermore, the rate of grade 3 or 4 treatment related adverse events is lower in patients receiving PD-1 blockade therapy versus ipilimumab which is similar to the decreased severity of autoimmune pathologies observed in PD-1 versus CTLA-4 knockout mice (169, 193). In contrast to PD-1 blockade antibodies, the αPD-L1 agent atezolizumab (MPDL3280A) has thus far received FDA approval only for urothelial bladder cancer and lung cancer (169, 209). Recently, studies have further complicated the role of PD-L1 by demonstrating that it binds to CD80 on T cells and provides another inhibitory signal (210). Thus, further studies are warranted to determine the role of PD-L1 in T cell inhibition in tumors and investigate which tumor types may benefit most from PD-L1 versus PD-1 blockade. A large number of clinical trials are currently underway targeting PD-1/PD-L1 as well as novel combination approaches (169). As previously mentioned, further study will be required to determine biomarkers of response to ICB and further mechanistic knowledge will be necessary to design effective combinatorial immunotherapies. Four clinical biomarker profiles for ICB treatment have already been proposed based on the presence of PD-L1 and TIL (211). The tumor are characterized as type I (PD-L1^+^TIL^+^), type II (PD-L1^−^TIL^−^), type III (PD-L1^+^TIL^−^), and type IV (PD-L1^−^TIL^+^) (211). In melanoma, where the data are most complete, the majority of patients are either type I (~38%) or type II (~41%). Type I patients are deemed to be the best responders to PD-1 blockade whereas type II tumors are estimated to have very poor prognosis due to their lack of immune cell infiltrates (211). Currently, the mechanisms that regulate the immune composition of a tumor are not well understood and there is a significant interest in treatments that can convert T cell non-inflamed (non-infiltrated) tumors to T cell inflamed (infiltrated) tumors (212).

#### Combinatorial Checkpoint Blockade

Despite the tremendous successes of ICB, to date, only a subset of patients achieve durable clinical responses (160, 167). However, the potency of immune checkpoint therapies has ushered in a new era of cancer treatment by offering the possibility of combining these drugs with conventional cancer treatments such as radiation, chemotherapy, and targeted molecular therapy (e.g., BRAF/MEK inhibitors). The prospects for such combination treatments in melanoma and other cancer types, as well as the clinical findings to date using such approaches have been expertly reviewed this year (213–215). The primary focus of this section will be to discuss the approaches involving combination checkpoint blockade therapies for melanoma that have demonstrated efficacy thus far. Nevertheless, it is pertinent to note that currently there are no clinical data to distinguish between ICB or BRAFi/MEKi targeted therapy as first line treatment for melanoma and a clinical trial (NCT02224781) is being conducted to provide direct comparisons between clinical outcomes in patients receiving checkpoint blockade drugs following targeted therapies and vice versa (215).

The success of combined ipilimumab and nivolumab has also been recently reported in a number of clinical trials. In 2015, Postow et al. reported the results of a study where previously untreated patients with metastatic melanoma received either ipilimumab in combination with nivolumab or with placebo preceding a subsequent treatment with nivolumab or placebo (216). The ORR was 61% in the combination treatment group versus 11% in the ipilimumab plus placebo group. Moreover, nearly 22% of patients treated with combination therapy achieved CR compared with none of the patients given ipilimumab and placebo (216). In the same year, results were published from a phase III trial in 945 patients with unresectable stage III or IV melanoma treated with nivolumab alone, nivolumab plus ipilimumab, or ipilimumab alone. The median PFS was 11.5 months for the combination group, 6.9 months for the nivolumab group, and 2.9 months for the ipilimumab group (217). However, serious (grade 3 or 5) treatment related adverse events in the combination treatment group were significantly higher reaching 55% compared with 27% for the ipilimumab group (217). These studies also indicate the superiority of combinatorial checkpoint blockade over monotherapy leading to the approval of ipilimumab and nivolumab dual therapy for melanoma in the USA, while its efficacy in other tumor types continues to be investigated (218). The successful use of combined checkpoint blockade has also sparked clinical interest in additional immune checkpoints some of which are undergoing preclinical or clinical investigation (167, 169, 218). A target of particular interest is the CD4 homolog lymphocyte activation gene-3 (LAG-3), which is expressed on Treg, effector CD4^+^ and CD8^+^ T cells, NK cells, B cells, and pDC and which also binds to MHC class II (167, 219). LAG-3 is an important negative regulator of CD4^+^ and CD8^+^ T cells and is required for Treg activity (219). The αLAG-3 antibody BMS986016 is currently being examined in a clinical trial (NCT01968109) for several advanced tumors both as a monotherapy and in combination with nivolumab (167). Another immune checkpoint that has exciting potential for tumor immunotherapy is TIGIT (T cell immunoreceptor with immunoglobulin and ITIM domain) (167). TIGIT is expressed by activated T cells, NK cells and is also expressed on highly functional subsets of Treg (219, 220). TIGIT has two ligands, namely, CD155 (poliovirus receptor, PVR) and CD112 (PVRL2) that are expressed on APC as well as on tumor cells (167). Likewise, TIGIT is reportedly expressed on TIL (219). The immunoregulatory functions of TIGIT are only recently beginning to be described (221). TIGIT can bind to CD155 on DC resulting in increased IL-10 and decreased IL-12 secretion (167). Ligation of TIGIT on Treg results in the expression of fibrinogen-like protein 2 (Fgl2), a Treg effector molecule that has broad immunosuppressive effects such as mediating Th1 and Th17 phenotype suppression in favor of Th2 (167, 222). In human melanoma, tumor-specific CD8^+^ T cells in peripheral circulation and CD8^+^ TIL were found to express both TIGIT and PD-1 and furthermore, TIGIT was upregulated in response to PD-1 blockade (223). Thus, the described functions of TIGIT further complicate our understanding of the immune response to αPD-1 treatment and provides further proof of the need of combinatorial approaches to overcome current barriers to ICB treatment. The positive results associated with ICB treatment have also renewed interest in a parallel treatment approach involving the development of agonistic antibodies for T cell costimulatory molecules such as CD137 (4-1BB), GITR (glucocorticoid-induced TNFR family related gene), and OX40 (CD134) many of which are currently undergoing clinical trials in combination with nivolumab (167, 169, 218). In 2016, early results were showcased for the antibody urelumab (αCD137) in combination with nivolumab (202). In melanoma, the ORR was observed to be 50% in patients who had not previously received checkpoint blockade therapy and was found to be independent of tumor PD-L1 status (202). Thus immune agonistic antibodies have revealed a plethora of novel possibilities for cancer treatment. Future studies will involve analyses of various combinations aimed at developing immunotherapies tailored to the specific tumor immune microenvironment (224).

### Adoptive Cell Therapy

Adoptive cell therapy involves the use of *ex vivo* manipulated cells transferred directly to patients to mediate antitumor immunity (50, 172). Thus far, the majority of clinical research in ACT has been conducted using autologous tumor-specific T cells (TIL) harvested and cultured from resected melanoma tissue (161, 173). Other cell types such as NK cells have also been investigated since the 1980s for their use in adoptive transfer therapy but have yet to be as widely studied as T cells (156). Thus, the primary focus of this section will be on studies with T cell ACT. The benefits of this approach are that it allows for the *ex vivo* expansion of tumor-specific cells that are not modulated by the immunosuppressive TME and can be administered in sufficient numbers to induce tumor regressions (50). As mentioned previously, this field was pioneered by Rosenberg and colleagues using autologous TIL from patients with metastatic melanoma and resulted in durable antitumor responses (186). Since that time, developments in molecular biology allowed for the elucidation of various tumor antigens and the development of genetically engineered T cell products with tumor-specific TCR or chimeric antigen receptors (CARs) (50, 225). To date, successful ACT through TIL transfer has been largely limited to melanoma although it is currently being studied in metastatic HPV-associated cancer and has been demonstrated to induce potent prophylactic clinical responses in HSCT recipients against Epstein–Barr virus-associated lymphoproliferative disorders (225). Lymphodepletion before TIL therapy has been shown to significantly augment clinical response, and although its precise mechanisms of action are not well understood, it is posited to complement TIL transfer by eliminating suppressive Treg and myeloid cells (50). In patients treated with autologous TIL therapy post lymphodepletion, the group of Rosenberg and colleagues at the NCI (Bethesda, MD, USA) has reported OR rates of 55% (226). These results are similar to those observed in patients from other centers that perform ACT using TIL such as MD Anderson (Houston, TX, USA) with an ORR of 48% in their patient cohort and Ella Cancer Institute (Raman Gat, Israel) with an ORR of 40% (50, 227). Overall, TIL therapy is not reported to be associated with severe adverse events, and the major toxic side effects are associated with the lymphoablative conditioning regimens (226). The primary hematological pathologies observed are anemia and thrombocytopenia necessitating transfusion in these patients, while patients in cohorts that receive TIL and IL-2 may report to develop grade 3 and 4 non-hematological toxicities (228). Currently, the predominant clinical form of ACT for melanoma is TIL therapy (50, 173). Nevertheless, there is also significant clinical interest in the use of highly specific T cells expressing TCRs specific to tumor antigens. These T cells can be generated through *in vitro* selection and expansion of specific antitumor clones (173). However, engineered T cells bearing conventional antitumor alpha beta TCRs or CARs have generated significant interest in the field of adoptive cell therapies (229). CARs are artificial receptors that were developed to circumvent the requirement of MHC–TCR interactions as many tumor cells downregulate MHC expression to escape the immune system (173). CARs consist of an extracellular ligand-binding domain constructed with immunoglobulin heavy and light chain variable regions fused through a transmembrane domain to intracellular CD3 zeta signaling chains in addition to CD28 or CD137 costimulatory domains for induction of complete T cell activation (50, 229). Currently, CAR T cells have demonstrated efficacy only in B cell malignancies using anti-CD19 CARs, to achieve response rates of up to 90% (173). However, a number of studies are currently underway investigating the use of CAR T cells in solid tumors (173). On the other hand, studies using transgenic tumor-specific TCRs have been tested in melanoma with the first proof-of-concept study being performed in 2006 using T cells transduced with a TCR against the melanoma differentiation antigen MART-1 (230). This early study showed evidence of clinical activity in only 2 out of 17 patients but a more recent report by Chodon et al. (231) demonstrated that MART-1 specific T cells in combination with MART-1 pulsed DC vaccine were able to induce tumor regression in 9 out of 13 studied patients (231). Thus, combining ACT with other immunotherapies may unveil potentially novel synergistic treatments that can overcome the current barriers to ACT. A number of clinical trials using ACT in conjunction with checkpoint blockade agents (nivolumab-NCT02652455) or targeted therapy (vemurafenib-NCT01659151) are being tested in patients with melanoma (173). A number of salient factors warrant consideration when discussing the merits of ACT immunotherapies for cancer. First, it is pertinent to mention that ACT requires *ex vivo* manipulation of cells, which is both expensive and labor intensive (173). Therefore ACT currently remains limited to a few specialized centers around the world (50). Furthermore, engineered T cells have the potential to induce stronger toxicities versus conventional TIL due to their clonal specificity toward a single antigen. This is a particular concern with TCRs targeted to antigens that are shared by tumor and normal tissue resulting in an immune activation versus the target but not necessarily against the tumor (on-target, off-tumor toxicity) (173). This effect has been observed in a number of trials. In a study treating patients with T cells bearing transgenic TCRs specific to MART-1 and gp100, several patients developed toxicities in the skin, ears, and eyes due to the presence of melanocytes in these organs (232). This effect has also been seen in other tumor types such as metastatic renal cancer where in a recent report, 4 out of 12 patients treated with CAR T cells specific to carbonic anhydrase IX (CAIX), developed liver toxicity due to the presence of this antigen in the bile duct (233). Thus, strategies will need to be developed to overcome such off-target effects of engineered lymphocytes and in the case of the aforementioned CAIX trial, hepatic T cell mediated toxicity was significantly lowered by treatment with blocking anti-CAIX antibodies (233). Although early studies showed that MART-1 and gp100 are among the major tumor antigens recognized by anti-melanoma TIL, recent advances in whole-exome sequencing offer the potential to reveal novel antigens (i.e. neoantigens) resulting from mutations that may be highly immunogenic but also safe due to their absence from the rest of the body (50). Another concerning immune-related toxicity observed in CAR and conventional T cell therapy is cytokine release syndrome, which presents as a systemic multisymptomatic inflammation causing fever, hypotension, and tachycardia (173). In terms of efficacy, a key concern using CAR T cells is that while they have shown remarkable results for hematological cancers, solid tumors are more difficult to treat and have a highly suppressive TME (173, 229). Nevertheless, advances in lymphocyte engineering have allowed for the conceptualization of a number of novel types of CAR T cells which can be switched on conditionally, or lack checkpoint molecules to prevent suppression. These novel CARs may have high utility for solid cancers and have been reviewed expertly elsewhere (229). Similarly, a novel type of molecule that has recently gained attention is a bispecific antibody construct that can bind to CD3 thus activating T cells as well as, a tumor antigen and is termed a bispecific T cell engager (BiTE^®^) (234). The anti-CD19 BiTE^®^ blinatumomab was approved by the FDA after showing activity in acute lymphoblastic leukemia but to date, none of the tested BiTE^®^ constructs tested in solid tumors have exhibited noteworthy antitumor responses (234). Novel developments in the field of genomic sequencing as well as T cell engineering have allowed for the conceptualization of highly personalized ACT treatment for cancer. Nevertheless, as discussed previously, without breakthroughs in *ex vivo* cell handling and automation, this therapy will remain highly costly and be limited to a few centers of excellence around the world.

### Cancer Vaccines

Vaccination for infectious disease represents a landmark of human medical achievement. Cancer vaccines seek to activate the immune system, in particular the T cells, to attack the tumor with the presentation of the tumor antigen in combination with an adjuvant (176). The vaccines may be univalent incorporating a single target antigen or polyvalent, consisting of allogeneic whole cells, or autologous tumor lysates (48). To date, none of the vaccine combinations tested in established tumors have shown the same efficacy as checkpoint blockade or ACT (165, 176). A number of studies have shown modest increases in clinical activity such as the study by Schwartzentruber et al. in 2011 that showed that patients with advanced melanoma treated with IL-2 and a gp100 peptide vaccine fared better than patients treated with IL-2 alone (median OS 18 versus 11 months, respectively) (48, 235). Nevertheless, cancer vaccination for solid tumors becomes particularly challenging due to the immunosuppressive TME and a constantly evolving tumor geared toward immune escape (165). In the past 30 years, as research unveiled the crucial role of DC in antigen processing and T cell activation, DC-targeted vaccines also became a major focus of cancer vaccination research (161). DC are considered to be ideal tools for inducing effective anticancer immunity due to their central role in antigen presentation and their ability to produce crucial effector cytokines (174, 236). The use of DC as anticancer vaccines has been comprehensively reviewed elsewhere (133, 145, 174, 237). Generally, this approach involves the generation of DC from isolated patient PBMC, which are then loaded with antigen and reinfused into the patient (161). Clinically a widely accepted DC maturation protocol involves the use of a cocktail containing TNFα, IL-1β, IL-6, and PGE2, resulting in the upregulation of MHC class I and II and costimulatory molecules (133). Other approaches in the clinic have used mixtures of prophylactic vaccines (which contain TLR agonists) containing Bacillus Calmette–Guerin (BCG)-SSI, Influvac, and Typhim (133, 238). DC maturation can also be induced by targeting the costimulatory receptor CD40 with CD40L (which is expressed by a range of immune cells but its most functionally important expression is on activated T cells *in vivo*) or anti-CD40 mAbs, resulting in the upregulation of costimulatory molecules and production of IL-12 (133, 237, 239). Currently, there is no gold standard in terms of maturation cocktails for DC and novel combinations continue to be tested both preclinically and in clinical trials (174). GVAX^®^ (Cell Genesys, San Francisco, CA, USA) are a cell product composed of irradiated autologous or allogeneic, tumor cells engineered to produce GM-CSF (240). GVAX^®^ vaccines were shown to elicit antitumor immune responses in a number of early clinical studies (241). However, a phase III trial using allogeneic GVAX^®^ in prostate cancer observed that this approach was not superior to current treatments (241). In melanoma, the GVAX^®^ approach has not shown significant clinical activity including a recent study by Lipson et al. that demonstrated that although melanoma GVAX^®^ was safely tolerated, it did not result in markedly increased anti-melanoma responses in peripheral blood T cells (175, 241). These early and currently ongoing studies demonstrate the difficulty of using cell-based approaches for cancer vaccination. Currently, Sipuleucel-T (Provenge^®^) is the only cell-based vaccine to be approved by the FDA for its observed clinically significant but modest increases in the OS of patients with prostate cancer (174). No such vaccine has yet received FDA approval for melanoma (161). In 2013, Carreno et al. reported the use of an autologous CD40L/IFNγ-matured DC vaccine pulsed with gp100-derived peptides and capable of producing IL-12 (242). In six out seven patients, this treatment successfully induced immune responses with three out of the six responding patients exhibiting tumor remissions (242). Despite these encouraging results, a number of concerns with cancer vaccination still exist, in particular with the choice of target antigen as tumors continue to continuously evade the immune response while novel mutated epitopes may not be sufficient for inducing potent antitumor T cell responses (161). Thus, there has been a significant clinical interest in the use of oncolytic viral vaccines for directly inducing cell death in tumors (48, 161). This approach attempts to harness the specificity of some oncolytic viruses for tumor cells as well as the induction of tumor cytolysis as an immune activating stimulus against non-infected tumor cells (177, 161). The first viral product to receive FDA approval is talimogene laherparepvec (T-VEC) which is a construct derived from herpes simplex virus 1 with deleted ICP34.5 and ICP47 genes and coding for human GM-CSF (177). In 2015, T-VEC was the first virotherapy that showed durable antitumor responses in patients with melanoma (178). Over 400 patients were treated with intralesional T-VEC or subcutaneous GM-CSF, and median OS was demonstrably higher in the T-VEC group versus the GM-CSF group (23 versus 19 months, respectively) (178). Moreover, the durable response rates and overall response rates were also higher in the T-VEC group than in the GM-CSF group with very limited toxicities associated with T-VEC treatment (178). As a result of these findings, the field of cancer vaccine research has been energized, and currently trials are underway to examine potential combination approaches using ICB in combination with oncolytic vaccine regimens to induce a long-lasting antitumor immune response (39, 161). The major limitation of the T-VEC approach is that it was found to be more effective in patients with less advanced (stage III and locally metastatic) melanoma than in patients with visceral metastatic disease (178, 161). Thus, in patients with established and advanced tumors, cancer vaccination approaches at best provide part of the solution for complete cure. With the complex immunoregulatory pathways that are established in advanced tumors, it may be difficult to achieve continued DC stimulation and activation through vaccines. Thus, a number of studies have begun to investigate the targeting of DC *in vivo* as crucial for the success for future immunotherapies (133). The success of T cell checkpoint therapy has already demonstrated the utility of treatments that mediate *in vivo* activation of antitumor immunity. Although a number of other cell types such as NK cells and MDSC have recently gained interest as targetable populations, DC remain a primary cell of interest for *in vivo* targeted immunotherapy due to their crucial roles as APC and in cytokine production (237, 243, 244).

### Nanoparticles as Multifunctional Immunotherapeutics

**Figure 3:**
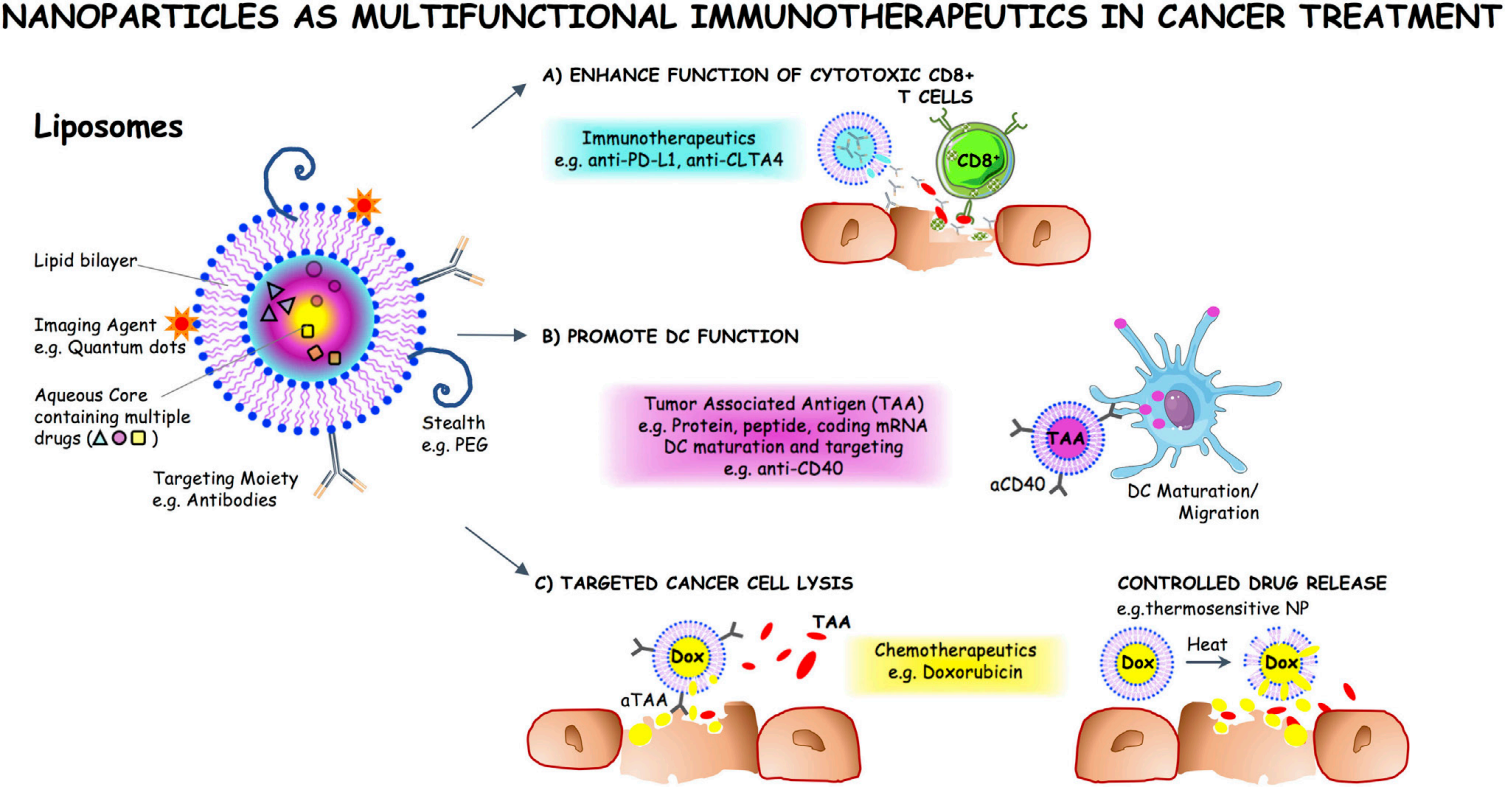
Multifunctional nanoparticles (NP) in cancer treatment. NP can be tailored to specific applications in tumor immunotherapy using versatile designs of various sizes, constituent biomaterials, and surface modifications. The surface of NP can be functionalized with specific polymers and antibodies to increase their targeting to certain types of cells. Liposomes are self-assembling nanosized vesicles comprised of phospholipids and cholesterol arranged in one or more lipid bilayers enclosing an aqueous core. NP such as liposomes can be used as platforms for the simultaneous delivery of multiple agents, such as **(A)** immunotherapeutics, e.g., anti-PD-L1 and anti-cytotoxic T lymphocyte antigen-4 (CTLA-4), to enhance the function of tumor-specific effector T cells; **(B)** tumor-associated antigens (TAA) and adjuvant targeted to dendritic cells (DC) to promote their function; **(C)** chemotherapeutics and targeted release thereof, for instance, using thermosensitive NP, to promote cancer cell death.

The past two decades have witnessed significant advances in our understanding of tumor immunology and the development of immunotherapeutic drugs (56, 163). In parallel, improvements in the field of nanomedicine provides us with a number of opportunities that can be used in combination with modern immunotherapies to enhance their antitumor efficacy (245–248). The primary advantage to NP is the supreme versatility in their design as their size, shape, constituent biomaterials, and surface modifications can be tailored for specific uses in tumor immunotherapy (Figure 3) (245, 247). Liposomes are self-assembling nanosized vesicles comprised of phospholipids and cholesterol arranged in one or more lipid bilayers enclosing an aqueous core (246, 249). Liposome-encapsulated drugs have been demonstrated to have reduced systemic toxicity profiles owing to improved pharmacokinetics and biodistribution (247, 249). Liposomal doxorubicin (Doxil) first received FDA approval in 1995, and even though it did not enhance OS, it is associated with improved toxicity profiles (247). This is of particular use for immunotherapy as many powerful adjuvants such as IL-2 and IFN-α have serious toxic side effects (161). In 2012, Park et al. demonstrated the utility of a biodegradable liposome and solid polymer hybrid gel as a dual delivery platform for IL-2 as well as an inhibitor of the immunoregulatory cytokine TGF-β (250). Treatment with this platform showed no significant toxicity in treated animals and more importantly delayed tumor growth was mediated via increased intratumoral NK and CD8^+^ T cell infiltration (250). Thus, NP can not only deliver drugs but also serve as platforms for simultaneous delivery of multiple agents. In the context of immunotherapy, NP can deliver tumor antigens, nucleic acids, and adjuvants (246, 248). There has also been research in the field of artificial APC NP platforms that present antigen loaded MHC I in combination with antibodies to the T cell costimulatory molecule CD28 (246). Finally, the surfaces of NP can be functionalized with specific polymers and antibodies to increase their targeting to certain types of cells (245). Even without direct targeting, systemically treated NP can accumulate at tumor sites due to “leaky” tumor vasculature (247). Earlier this year, Koshy et al. reported the antitumor potency of liposome-encapsulated cGAMP (251). The authors showed that cationic liposome loaded with cGAMP resulted in passive lung-specific delivery in metastatic B16F10 melanoma lung tumors leading to pronounced antitumor activity and the formation of immune memory (251). Currently, a number of unique immunotherapeutic NP are being investigated in Phase I–III clinical trials (247). However, to date no directly DC-targeted NP formulation has reached clinical trials. As DC play central roles in priming antitumor immunity as well as directly influencing the immune infiltration of T cells into cancer (212), NP targeted to DC warrant inclusion in future combinatorial immunotherapies (252). In 2016, Kranz et al. developed a strategy to deliver RNA-NP to DC in a pilot study with three melanoma patients (105). The RNA encoded for the melanoma antigens NY-ESO-1, MAGE-A3, tyrosinase, and TPTE (transmembrane phosphatase with tensin homology) and resulted in IFNα and antigen-specific T cell responses in all three patients (105). This approach was administered systemically and was not found to be associated with any adverse effects. This study thus opens a new field of DC-targeted, highly potent immunotherapies for cancer. NP are biodegradable, relatively cost-effective (compared with *ex vivo* manipulated cells) (133) and highly multifunctional platforms for enhancing modern immunotherapies or developing independent DC-targeted treatments (247).

## Summary

Currently, the field of immunotherapy is one of the most promising avenues of research in the quest to develop long-term broadly acting treatments for cancer (55, 161, 253). The possibilities for synergistic combinations with radiation, chemotherapy, and small molecule targeted treatments have also unveiled countless possibilities for tailoring individualized therapies in the drive towards “precision medicine” (213, 214, 254). However, evolutionary checkpoints against autoimmunity and the fact that cancer arises from self-tissue presents a particularly challenging landscape for developing multitargeted immunotherapies that are cost-effective, safe, and efficacious. Conceptually, there are four general facets of tumor immunity that must be achieved for successful immunotherapy (253). These are the removal of immunosuppressive cues, the induction of immunogenic cell death in tumors, improved activity of APC and increased T cell effector functions (253). In addition to a comprehensive overview of the immune contexture of a tumor, other host specific factors such as genetics and individual microbiota must be further dissected to determine their interplay with immunotherapeutic agents (255). In recent years, advances in high-throughput techniques such as next-generation sequencing and mass cytometry (CyTOF) have enabled highly detailed phenotyping of cancer (256, 257). However, there is still an unmet need for bioinformatics platforms and deep-learning algorithms that can assist biologists with mining and analyzing such massive datasets (258). Finally, due to the need to finely target various facets of tumor immunology in immunotherapy, NP technology may become indispensable as the delivery vectors and the platforms upon which these multifunctional therapeutics are designed (248).

## Author Contributions

MS conceptualized the manuscript and oversaw all aspects of its completing including writing, figure design, and literature review. HS and TG contributed equally to this manuscript by performing literature review and writing of the manuscript. RH provided medical expertise in the subject matter during writing of the manuscript and contributed clinical images.

## Conflict of Interest Statement

The authors declare that the research was conducted in the absence of any commercial or financial relationships that could be construed as a potential conflict of interest. The reviewer KC and handling editor declared their shared affiliation.
